# Dynamic Excitatory and Inhibitory Gain Modulation Can Produce Flexible, Robust and Optimal Decision-making

**DOI:** 10.1371/journal.pcbi.1003099

**Published:** 2013-06-27

**Authors:** Ritwik K. Niyogi, KongFatt Wong-Lin

**Affiliations:** 1 Gatsby Computational Neuroscience Unit, University College London, London, United Kingdom; 2 Intelligent Systems Research Centre, University of Ulster, Magee Campus, Londonderry, United Kingdom; University of Oxford, United Kingdom

## Abstract

Behavioural and neurophysiological studies in primates have increasingly shown the involvement of urgency signals during the temporal integration of sensory evidence in perceptual decision-making. Neuronal correlates of such signals have been found in the parietal cortex, and in separate studies, demonstrated attention-induced gain modulation of both excitatory and inhibitory neurons. Although previous computational models of decision-making have incorporated gain modulation, their abstract forms do not permit an understanding of the contribution of inhibitory gain modulation. Thus, the effects of co-modulating both excitatory and inhibitory neuronal gains on decision-making dynamics and behavioural performance remain unclear. In this work, we incorporate time-dependent co-modulation of the gains of both excitatory and inhibitory neurons into our previous biologically based decision circuit model. We base our computational study in the context of two classic motion-discrimination tasks performed in animals. Our model shows that by simultaneously increasing the gains of both excitatory and inhibitory neurons, a variety of the observed dynamic neuronal firing activities can be replicated. In particular, the model can exhibit winner-take-all decision-making behaviour with higher firing rates and within a significantly more robust model parameter range. It also exhibits short-tailed reaction time distributions even when operating near a dynamical bifurcation point. The model further shows that neuronal gain modulation can compensate for weaker recurrent excitation in a decision neural circuit, and support decision formation and storage. Higher neuronal gain is also suggested in the more cognitively demanding reaction time than in the fixed delay version of the task. Using the exact temporal delays from the animal experiments, fast recruitment of gain co-modulation is shown to maximize reward rate, with a timescale that is surprisingly near the experimentally fitted value. Our work provides insights into the simultaneous and rapid modulation of excitatory and inhibitory neuronal gains, which enables flexible, robust, and optimal decision-making.

## Author Summary

Perceptual decision-making involves not only simple transformation of sensory information to a motor decision, but can also be modulated by high-level cognition. For example, the latter may include strategic allocation of limited attentional resources over time in a decision task to improve performance. At the neurophysiological level, there is evidence supporting attention-induced neuronal gain modulation of both excitatory and inhibitory cortical neurons. In the context of perceptual discrimination tasks performed by animals, we make use of a biologically inspired computational model of decision-making to understand the computational capabilities of such co-modulation of neuronal gains. We find that dynamic co-modulation of both excitatory and inhibitory neurons is important for flexible, and cognitively demanding decision-making while also enhancing robustness in the decision circuit's functions. Our model captures the neuronal activity and behavioural data in the animal experiments remarkably well. Decision performance in a reaction time task can be optimized, maximizing the rate of receiving reward by using fast gain recruitment. Our experimentally fitted timescale is near the optimal one, suggesting that the animals performed almost optimally. By providing both computational simulations and theoretical analyses, our computational model sheds light into the multiple functions of rapid co-modulation of neuronal gains during decision-making.

## Introduction

Perceptual decision-making often requires temporal integration of sensory information and its subsequent transformation to a categorical motor choice [1]. The decision or response times in speeded perceptual decision tasks can range from tens of milliseconds to a second or more [2], [3]. Perceptual decision-making is not a simple standalone feed-forward sensorimotor integration process, but is distributed and subjected to various neuromodulatory or cognitive control processes, possibly to enhance decision performance [1], [4]–[12].

In difficult sensory discrimination tasks, for example, with near ambiguous stimuli, the optimal approach would be to integrate sensory evidence over a long period of time. However, long temporal integration is seldom observed in experiments, and there is evidence suggesting a temporally increasing urgency signal during decision formation [13]–[21]. Neural correlates of such urgency signals have been found in parietal cortical neurons [12], [17]. In other studies, the parietal and extrastriate cortical areas are found to exhibit gain modulation of neuronal firing rates which are dependent on behavioural context and attention [22]–[31], and various biophysical mechanisms of neuronal gain modulation have been proposed [23], [24], [32]–[51]. Visual attention in the parietal cortex has been studied as an integral component in perceptual decisions [30]. Interestingly, visual attention seems to be capable of simultaneously modulating both excitatory and inhibitory cortical neuronal gains [52], and can have a time-varying nature [53]

Previous computational and theoretical models have incorporated urgency signals or dynamic gain modulation in their decision-making models [12]–[20], [54]. However, most of these models are largely abstract and hence do not allow incorporating inhibitory neuronal gain modulation. Thus, the computational capabilities of simultaneous modulation of excitatory and inhibitory neuronal gains on perceptual decision-making dynamics and behaviour remain unclear.

In this work, we incorporate a time-varying excitatory-inhibitory gain modulation mechanism into our previous cortical microcircuit model of decision-making [55], [56]. The model is based on and compared very closely to two classic visual motion discrimination task experiments performed in animals: a reaction time task and a cued response task [57]. Our model is constrained by both the neuronal and behavioural data of a reaction time task. The model naturally captures the essential characteristics of the neuronal firing rates throughout a trial in both experiments, with weaker gains in the seemingly less cognitively demanding cued response task. Dynamical systems analysis is used to provide insights into the flexibility and robustness of the network dynamics under the co-modulation of neuronal gains. We also show that with gain modulation, strong recurrent synapses are not necessary for making and storing decisions. Finally, using realistic temporal delays in the reaction time task, our model simulations show that rapid recruitment of gains can optimize decision performance, and suggests that the animals may adopt such a strategy. Part of this work has been presented at the Computational and Systems Neuroscience 2010 meeting [58].

## Results

### Two-choice motion-discrimination tasks

**Figure 1:**
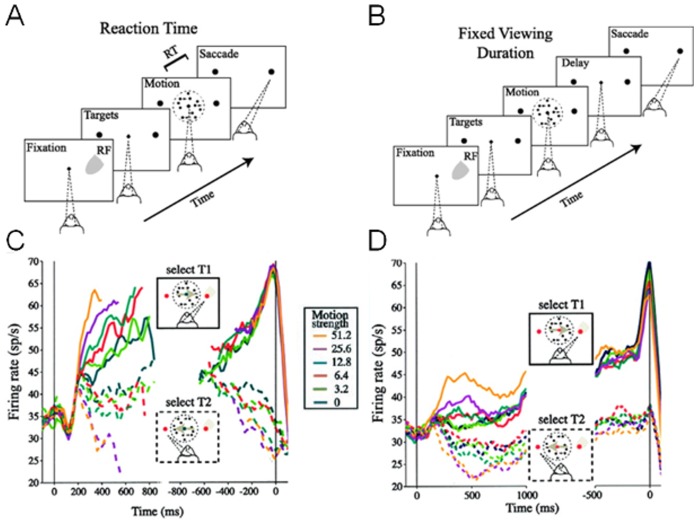
Dot-motion discrimination task and neural recording data. (A,B) Sequence of time epochs within a trial in the reaction time (RT) task (A) and the fixed duration (FD) task (B). The trial begins with the appearance of a fixation point followed by two choice targets, and then a motion stimulus in the form of computer-generated random dots. The motion stimulus has a fraction of the dots moving towards either the left or the right choice target, constituting the motion coherence of the stimulus. The subject is trained to discriminate this motion coherence and make a motor choice (saccade) in the same direction as this motion coherent direction towards the corresponding choice target (right in the above figure). In the RT task, the subject makes a saccade once it has accumulated sufficient evidence in support of its decision, and the motion stimulus is removed once a saccade is made. In the FD task, the motion stimulus is presented for a fixed duration of time (e.g. 1 second) before it is removed during a delay period. The subject has to remember the motion coherent direction to guide its saccadic choice. (C,D) LIP neural firing rate timecourse from the RT task (C) and the FD task (D). Dashed (bold) lines are neural activities with eventual saccade moving away from (towards) their response fields, T2 (T1). Reproduced with permission from [57].

The classic experiments of [57] involved primates performing two versions of a dot-motion-discrimination task. In the reaction time (RT) task (Figure 1A), subjects were trained to make a (e.g. left- or rightward) saccadic eye movement in the direction of the motion coherence of the random dots stimulus, at their own pace. The stimulus was presented till a saccade was detected. For the fixed duration (FD) task (Figure 1B), they were instead allowed a (1 sec) fixed viewing duration following which they were required to withhold their decision response until a cue was given to respond. Neuronal firing activity in the lateral intraparietal area (LIP) and behavioural performance were simultaneously recorded (Figure 1C,D).

### A biological cortical circuit model for decision-making

**Figure 2:**
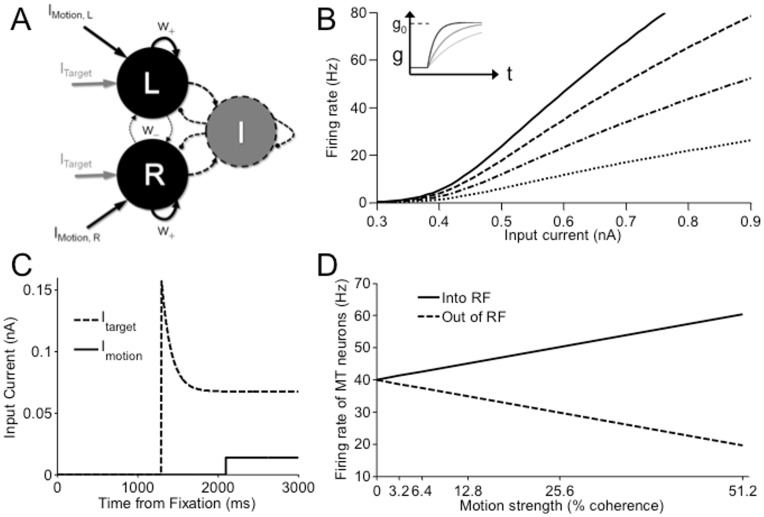
A decision-making model. (A) Network model architecture. The network is fully connected, with recurrent inhibition provided by an implicit population of inhibitory interneurons (dashed lines). Inputs to the left- or right-motion selective population of LIP neurons include that from upstream MT/V5 neurons (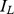 or 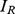) and the choice targets (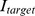). (B) Sample gain modulation on a single-cell input-output relation. Dotted to solid curves show effect of increasing gain. Inset: Temporal evolution of gain, light to dark colours show decreasing time constant of gain modulation. (C) Timecourse of input currents. (D) Firing rate of an upstream MT neuron encoding motion stimulus, when motion is into or out of its response field (RF).

We used a neural circuit model of decision-making [55] that consists of two competing excitatory neural populations, each selective to a presented stimulus, e.g. with opposite motion direction selectivities in a motion discrimination task. An implicit population of interneurons provides common inhibitory feedback in the network (see Figure 2A). This model was reduced and approximated from a spiking neuronal network model of 2000 neurons [59] to an effectively analyzable model.

#### Input synaptic currents and output firing rates

As in [55] and [56], the input-output function of a single noisy excitatory cell is
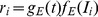
(1)where 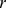 is the population-averaged firing rate, 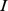 is the total synaptic input current to a neuron, and 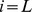 or 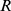, denoting selectivity to a leftward or rightward motion stimulus, respectively. The non-linear input-output function 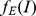 is approximated from the first-passage time input-output relation of a leaky integrate-and-fire neuron [46], [55], [60] (see Eq.(9) in the *Materials and Methods* section). 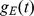 and 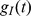 represent the time-varying gain modulation parameter (see Figure 2B) of excitatory and inhibitory cells, respectively. For the inhibitory interneuronal population, we assume that 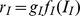 is linear so that it can be implicitly embedded in the reduced two-variable model for analysis [55].

Following [56], we assume that recurrent excitation and inhibition in the network is mediated through NMDA and GABA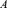 receptors, respectively. At any given time, the total synaptic currents to the two neural populations are given by
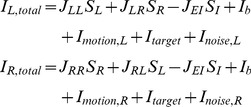
(2)where the 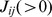 represents the effective synaptic coupling from neural pool 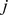 to 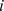. Within a selective population 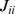 is a constant ( = 0.32 nA) times the (dimensionless) strength of the recurrent excitation, 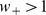, while between selective populations, 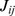, is the same constant times 
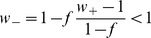
, where 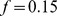 is the fraction of selective neurons. 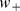 (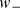) can be viewed as representing synaptic potentiation (depression) between neurons in the same (different) excitatory population after learning [61], [62]. Unless otherwise specified, we used 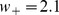, as in [56].


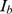
, 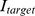, 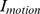 and 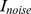 are the input currents due to overall background inputs from neurons outside the local network, static choice targets within the response fields of the LIP neurons, output of upstream motion selective MT/V5 neurons, and noise from the motion stimulus and from within the brain, respectively (Figure 2A, C). 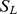 and 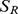 are synaptic gating variables of NMDA-mediated receptors, i.e. the population-averaged fraction of open channels. 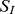 is the gating variable of GABA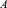 receptors.

Following the deduction of [55], the network can be further reduced to a two-variable model, as we may consider
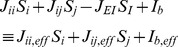
(3)with 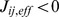 allowing competition via effective mutual inhibition between the two selective excitatory populations (see Eq.(10) in the *Materials and Methods* section).

#### Choice targets and motion stimulus inputs

The input current due to the encoded target (Figure 2C) can be modeled as
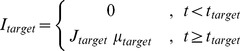
where 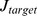 is the coupling strength for the choice target and 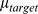 is the firing rate of upstream visual areas encoding the target. We set the choice target onset time, 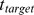 to be at 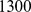 ms after the start of a trial simulation. The firing rate of upstream neurons encoding the target, 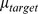 first attains 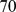 Hz before exponentially decaying over time to 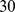 Hz with a time constant of 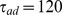 ms. The exact exponential time course, which is adopted from [56], is not important for our model's computations, but follows the experimental data in [17], mimicking visual adaptation of the stationary visual target stimuli.

Firing rates of upstream MT neurons, selective for a particular direction of motion, can be assumed to increase (decrease) roughly linearly with the motion strength, when the motion is in the preferred (anti-preferred) direction of the neuron, in the regimes of motion strength experimentally tested [63] (Figure 2D). For simplicity, we assume that there are no differences between the slopes of this linear function, except in sign, for motion in the preferred or anti-preferred directions (our results are qualitatively similar if we assume lower slopes, e.g. 2–3 times shallower, for the anti-preferred direction, see Figure S4). Thus the external current encoding motion stimulus relayed to the LIP neurons is expressed as
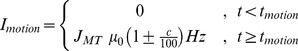
where 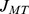 is the coupling strength for the motion stimulus, 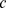 ranging from 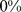 to 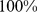 represents motion coherence of the random dots, and 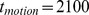 ms is the motion stimulus onset time. The 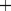
(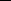) signifies motion direction in the preferred (anti-preferred) direction of the neuron 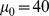 Hz corresponds to the mean firing rate of MT neurons for the ambiguous zero motion coherence (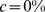 [63]).

#### Time-varying gain modulation

We assume the simplest form of gain modulation, a “gain field” [24], [46], [64] 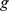 with an effective time constant and amplitude (Figure 2B inset; cf. sigmoidal function in [65], hyperbolic function in [17], and the more complex function in [15], [16]):
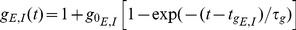
(4)where 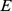 or 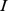 denotes the excitatory or inhibitory population. 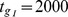 ms is the onset time of gain modulation of inhibitory neurons while 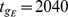 ms is that for excitatory neurons. The 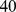 ms onset delay is used to replicate the signature ‘dip’ phenomenon [18]. We then chose a time constant of 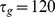 ms in our simulations, to fit the neuronal and behavioural data of [57]. This is also around the timescale of rapid covert shift of attention in area LIP [66]. Our results are independent of the specific neural implementation of the gain modulation mechanism.

Setting 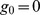 would lead to a gain factor of 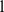, which we assumed throughout the pre-motion stimulus epoch in a trial. In a RT task, upon motion stimulus onset, 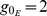 and 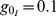. In a FD task, the gains were scaled to 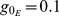 and 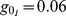 during motion stimulus presentation to keep the firing rate encoding the accumulated decision below the fixed response threshold. However, upon cue to respond (at 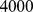 ms), the gain was increased to that of the RT task (with the same time constant). The specific values of 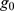 were selected to fit the experimental data. The increasing gain over the course of a trial during motion stimulus presentation is representative of the fact that attentional modulation of decision-making would increase over the course of evidence accumulation, creating an urgency-to-response signal during this process.

Note that while representing the gain modulation of the explicit excitatory selective populations is straightforward (see Eq. 1), the effect of changing the gain of the inhibitory interneurons implicit in our effectively two population model is subtler. Increasing the inhibitory gain decreases the effective synaptic couplings in 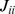 and 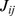 by a linear factor of this gain as well as decreasing the mean background synaptic input 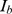 by another linear factor (see Eq. 11 in the *Materials and Methods* section).

#### Dynamical equations

The slowest decay time constant in the model is that of NMDA receptors (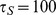 ms). All other dynamical variables operate at a much faster timescale and are assumed to achieve their steady states relatively rapidly. Thus the dynamical equations governing the network are [55], [56]

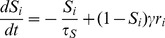
(5)where 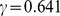 is a fitted parameter [55].

#### Model parameters

In any parameterized model, there is always a fine balance between incorporating more biological details and reducing the number of model parameters. There are certainly simpler models than ours, with minimum parameters, such as the drift-diffusion model, which, with or without gain modulation can model the behavioural data [15], [16], [67], [68]. However, as stated earlier, these abstract models cannot directly correlate with neuronal and synaptic properties, and thus cannot realistically incorporate inhibitory gain modulation.

Other than the new parameters pertaining to our gain modulation mechanism, the entire model parameters and their values are identical to those in our previous modelling work [55] and [56], more directly following the latter. The list of the more critical adopted and new parameters is shown in Tables 1 and 2, respectively.

**Table 1:** Parameters based on previous work: ^1^Wang (2002), ^2^Wong and Wang (2006), ^3^Wong et al. (2007), ^4^Eckhoff et al. (2011), ^5^Churchland et al. (2008), and ^6^Britten et al. (1993).

Parameter	Interpretation	Value	Reference
*τ_s_*	(NMDA-mediated) synaptic gating variable time constant	100 ms	[1], [2], [3], [4]
*τ_noise_*	(AMPA-mediated) synaptic time constant	2 ms	[1], [2], [3], [4]
*τ_ref_*	LIP neuronal (absolute) refractory period	2 ms	[1], [4]
*τ_ad_*	LIP neuronal adaptive time constant	120 ms	[3], [4], [5]
*W*+	Synaptic potentiation factor within selective population	2.1	[2]
*f*	Fraction of selective neurons in excitatory neurons	0.15	[1], [2], [3], [4]
*J_LL_*, *J_RR_*	Recurrent synaptic strength within a selective population	0.32 nA	[3]
*J_LR_*, *J_RL_*	Recurrent synaptic strength between selective populations	0.32 nA	[3]
*J_EI_*	Synaptic strength from inhibitory to excitatory populations	8.58 nA	[3]
*J_IE_*	Synaptic strength from excitatory to inhibitory populations	0.32 nA	[3]
*J_target_*	Synaptic strength for choice target	0.0022 nA/Hz	[3]
*J_MT_*	Synaptic strength for motion stimulus	0.000225 nA/Hz	[3]
*μ_0_*	MT firing rate for zero motion coherence	40 Hz	[1], [6]
*σ_noise_*	Standard deviation of noise	0.015 nA	2, 3

**Table 2:** Parameters for fitting ^A^neural and ^B^behavioural data in current work.

Parameter	Interpretation	Value	Data types to fit	Notes
*g_0E_*	Excitatory gain maximal amplitude	0 (f and t); 2 (m) in RT; 0.1 (m, d) in FD, 2 (c) in FD	A, B	Explored in Results section
*g_0I_*	Inhibitory gain maximal amplitude	0 (f and t); 0.1 (m) in RT; 0.06 (m, d) in FD, 0.1 (c) in FD	A, B	Explored in Results section
*τ_g_*	Gain modulation time constant	120 ms	A, B	Explored in Results section
*t_gI_*	Inhibitory gain onset time	50 ms after m onset	A (‘dip’ behavior)	
*t_gE_*	Excitatory gain onset time	90 ms after m onset	A (‘dip’ behavior)	

RT: reaction time task; FD: fixed duration task. f: fixation; t: fixation and target epoch; m: fixation, target and motion epoch; m, d: fixation+target+delay period in FD task; c: cued to response in FD task.

The new parameters, pertaining to excitatory and inhibitory gain modulation consist of only their onset times (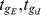), time constants (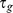), and amplitudes (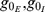). These parameters were used to provide a qualitative rather than quantitative fit to the neural and behavioural data, and we simulated predictions at a range of parameter values until the desired fits were isolated. The onset times are constrained to qualitatively but reliably replicate the signature dip phenomenon of the firing rates right after motion stimulus onset and are fixed throughout this study. 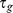 allows the network to dynamically change configuration from the dip period to the motion stimulus period. The value of 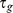 is obtained from fitting the neuronal and behavioural data and we shall explore its effects towards the end of the paper. It also affords us an opportunity to study the optimal time-scale of gain recruitment in RT tasks, similar to [54], but with a more realistic model and simulation setting (the time parameters in the simulated experimental trial follow closely that of [57] and [15]). The model's ability to form and store decisions is independent of the values of 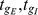 and 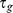. This depends on the gain amplitude parameters (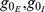). We shall show that our model is not sensitive to our chosen values of the gain amplitude parameters 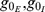. The details of how the new parameters were constrained are provided in the *Materials and Methods* section and in Table 2.

#### Epochs in a trial

A simulated trial can be categorized under separate epochs [17] (i) fixation-only (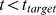), with baseline firing rates; (ii) fixation-target (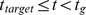), with an initial phasic burst of activity and then adapting into a steady firing; (iii) fixation-target-gain (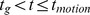), where the neuronal gains are assumed to start increasing; (iv) fixation-target-gain-motion (
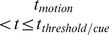
) with an additional random-dot motion stimulus, and firing rates of the two selective populations start to deviate. 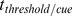 is the time of threshold crossing when the motor action of a saccade is initiated in an RT task or the time of cue presentation in the FD task.

A motor action (saccadic eye movement) is triggered when the higher population firing rate crossed a prescribed threshold at 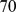 Hz (cf. Figures 7 and 9 in [57]). In a RT task, the time elapsed from motion onset to threshold crossing yielded the decision time. A non-decision latency of 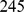 ms due to sensory signal transduction and motor saccadic preparation was added to the decision time to obtain the observable RT.

### Excitatory-inhibitory gain modulation in the reaction time task

**Figure 3:**
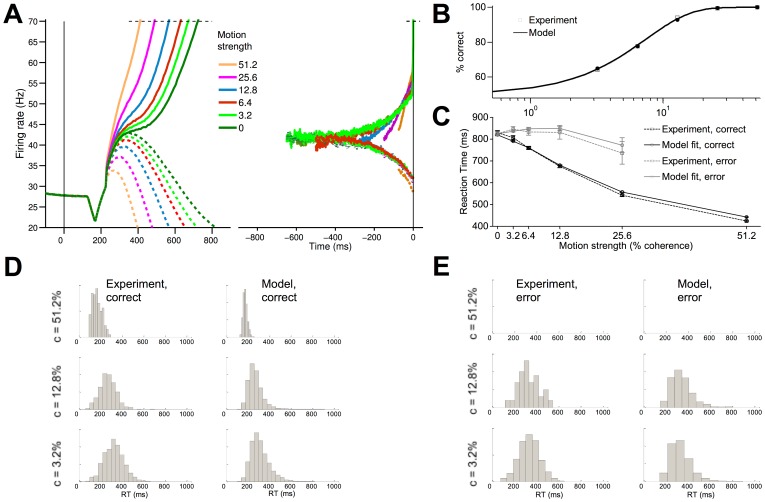
Network model reproduces neural and behavioural data in the RT task. (A) Activity timecourse of model, averaged over multiple trials, with different motion coherences, locked to motion onset (left) or saccadic response (right). Response threshold at 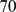 Hz, compare with Figure 1. (B,C) Accuracy (B) and mean RT (C) generated by model and in the experiment of [57]. Psychometric functions fitted with a Weibull function, 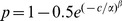:, where 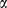 is the discrimination threshold at which performance is at 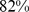 correct, and 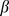 yields the slope of the psychometric function. 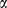 and 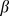 of the model (experiment): 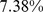 and 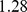 (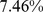 and 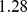), respectively. Error bars denote standard errors. (D,E) RT distributions generated by model and in the experiment for correct (D) and error (E) trials. Upper, middle, lower panels: 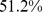, 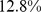, 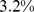 coherence, respectively.

As shown in Figure 3A our model captures the essential timecourse of the neural data in a RT task. Similar to previous models [55], [56], [59], our model reproduces the faster ramping up (down) of firing rates for larger motion coherences when the motion is into (out of) the response field of a LIP neuron. Our model can also reproduce the psychometric (accuracy) and chronometric (reaction time, RT) data of the experiment of [57] (Figure 3B and C), and the RT distributions for both correct and error trials (Figure 3D and E). In addition, our model naturally captures the characteristic dip at motion onset by modulating inhibitory gain before excitatory gain, providing an alternative mechanism to our earlier work [56]. In the RT task experiments [57], the firing rates of LIP neurons during motion stimulus presentation were observed to diverge from a level higher than the adapted firing rate during the target epoch (Figure 1C). Our model is able to replicate this phenomenon, unlike previous work [56], [69], [70], [71].

**Figure 4:**
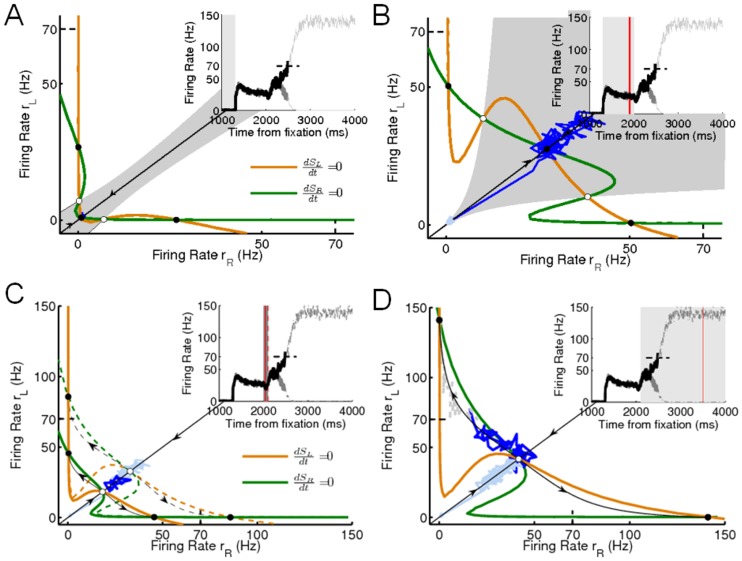
Phase-planes at different epochs of a trial in the RT task. (A) Fixation (pre-target onset): 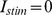, 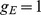, 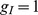. Orange and green curves represent nullclines: where 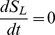 and 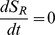, respectively. (B) Fixation with targets: 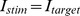, 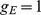, 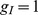. Grey regions show the basin of attraction for the symmetric (on-diagonal) attractor (see text for definition). (C) Fixation, with targets and gain onset: 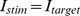. Solid (dashed) nullclines: 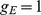, 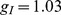 (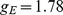, 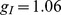). Solid nullclines: with inhibitory gain onset prior to excitatory gain onset; dashed nullclines: with both inhibitory and early stage of excitatory gain increase. (D) Fixation, with targets, gain modulation, and motion stimulus (zero coherence): 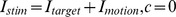, 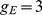, 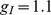. Closed and open circles represent stable (attractors) and unstable steady states, respectively. Diagonal line (stable manifold) and the curve to which off-diagonal trajectories near an unstable steady state are repelled to (unstable manifold), are shown in black and denoted by arrows moving towards or away from the unstable steady state, respectively. Blue: a sample trial with the corresponding epochs in a trial in bold and labeled in inset, grey lines denote the unobserved part of the simulation trial after saccade initiation. Red: denotes the time when the phase plane was viewed. Note the different scales between the top and bottom panels.

This activity timecourse of our two variable (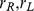) model can be better understood by investigating its dynamics on a two dimensional phase/state space called the phase plane (see *Materials and Methods*, Figure S1 and Figure 4). Here we shall for simplicity consider only the unbiased motion stimulus (i.e. zero motion coherence, 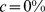) condition. We defer the explanation for non-zero motion coherence, for both correct and error trials to [55], [56], and [72].

Following the procedure as in previous work [55], , we first set both dynamical equations in Eq. (5) to be 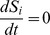 and solve for the 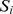
's, where 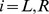. The solutions for each equation (called a nullcline, orange and green curves in Figure 4) can be plotted in the two dimensional (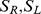) phase plane. We transform the dynamical variables (synaptic gating variables) 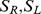 to firing rates 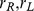 (see *Materials and Methods*), enabling direct comparison with the experimental data.

Intersections of the nullclines, by definition, give us the steady states of the network. Steady states can either be stable, i.e. attractors (black filled circles in Figure 4), or unstable (open circles in Figure 4, see *Materials and Methods* and Figure S1). In the firing-rate space, a steady state can be symmetric, i.e. lie along the phase plane diagonal, or asymmetric, i.e.. off-diagonal. This means the firing rates of the competing selective populations can be equal (symmetric) or unequal (asymmetric).

Symmetric (on-diagonal) attractors allow stable, steady, equal firing rates of both selective populations, which prevents decision-making and categorical choice. A symmetric unstable steady state on the other hand can force the network's state to move off-diagonally, causing the firing rate of one (winning) population to increase while that of the other (losing) to decrease, enabling decision-making and categorical choice. Asymmetric, ‘choice’ attractors ensure that the firing rates of the winning (losing) populations reach a stable steady state, and do not increase (decrease) without bound. Our modus operandi then is to let the network reach symmetric attractors during the fixation and fixation-target periods and then using gain modulation, approach (along the diagonal line) a symmetric unstable steady state during the fixation-target-gain-motion period (see Movie S1).

During the fixation period (Figure 4A, inset, shaded region), the firing-rates of both competing selective populations are at a low, spontaneous/baseline stable state. This is represented by the network starting off and remaining at a low symmetric attractor (Figure 4A).

When the choice targets appear, a burst of input current (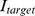) transforms the nullclines such that there is only one attractor, which is symmetric (Figure S1A, Movie S1). This precludes any winner-take-all dynamics and allows the firing rates of both competing populations to be reliably activated to an equal, high level. After adaptation, the network settles at a symmetric attractor (Figure 4B) with a higher firing-rate than that during the fixation period. The grey region of Figure 4B represents the *basin of attraction* of this symmetric attractor. Trajectories starting in this region are attracted into this symmetric attractor. Although additional asymmetric attractors are present, the large basin of attraction of this symmetric attractor shows that this symmetric attractor is very stable. Even with noise (see noisy trajectory in Figure 4B (dark blue)), any winner-take-all dynamics and consequently, any decision-making during the target period is prevented, and both populations fire at the same rate prior to the onset of the motion stimulus (Figure 4B, inset, shaded region), consistent with experimental data [17].

So far, we have ensured that our model behaves similar to our previous work [55], [56], although later, we shall show that the presence of multiple stable states during the fixation and fixation-target periods is not necessary. However, the model starts to differ from here onward. Immediately upon motion stimulus onset, the gain of the inhibitory neural population is turned on, creating a lower but nearby symmetric, unstable steady state (Figure 4C, solid nullclines). Only trajectories starting on the diagonal line (called the stable manifold, see *Materials and Methods*, and represented by the black curve with arrows pointing towards this symmetric unstable steady state) are attracted to this unstable steady state, all others are repelled away. Since the attractor formed after target-adaptation is symmetric, i.e., on diagonal (Figure 4B), the network starts from and moves along the diagonal line (with equal firing rates) towards this lower unstable steady state (sample trajectory in Figure 4C, dark blue). This creates an equal ‘dip’ in firing rates. Before it can reach the unstable steady state, the gains of the excitatory selective populations are activated. Consequently, the symmetric unstable steady state is raised (Figure 4C, dashed nullclines), and the population firing rates increase, once again along the diagonal line (with equal firing rates). This yields the recovery from the ‘dip’. Our delayed gain onset can thus create the dip phenomenon and the recovery from it (Figure 4C, inset; Figure 3A) without lowering the overall inputs to the system (as implemented in previous modelling work).

The input current due to the motion stimulus (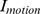) causes the net input to the network to increase, causing the symmetric unstable steady state (after momentarily becoming stable, see Movie S1) to be at an even higher firing rate (Figure 4D). As we shall show, the co-modulation of both excitatory and inhibitory gains is necessary to allow this symmetric unstable steady state to be at a higher activity level than the adapted target firing-rate (compare with Figure 4B). After briefly moving towards the symmetric unstable steady state (sample noisy trajectory in Figure 4D, dark blue), the network eventually gets perturbed off the diagonal line. The network is then repelled away from this unstable steady state to another curve (called the unstable manifold, see *Materials and Methods*, and shown by the black curve with arrows pointing away from the symmetric unstable steady state) and towards one of the asymmetric ‘choice’ attractors. This causes the firing rates of the competing selective populations to diverge such that the firing-rate of one population ramps up while that of the other ramps down, exhibiting winner-take-all behaviour, and forcing a decision. Prior to reaching one of these choice attractors, a motor action (saccade) is made when the network crosses the motor/saccadic threshold (70 Hz in our case; dashed horizontal black lines in all panels and insets of Figure 4). The various epochs within a trial are summarized in Movie S1. Additionally, the network can be reset before the start of the next trial by allowing the gains to decay to a low value after the threshold is crossed (see Figure S2).

### Robust and flexible decision dynamics with excitatory-inhibitory gain modulation

Having accounted for the observed neural and behavioural data, we shall now demonstrate how gain modulation of both excitatory and inhibitory neurons is necessary for flexible and robust decision-making. For simplicity, we shall consider an unbiased stimulus input, i.e. zero motion coherence (see [72] for biased stimulus input) condition.

Following [55], we will map out the range of possible stable and unstable steady states, i.e. the stability (bifurcation) diagram of the system, as a function of a variable or parameter of interest. Parameter regimes where both asymmetric attractors and symmetric unstable steady states exist are regimes of decision-making and categorical choice.

**Figure 5:**
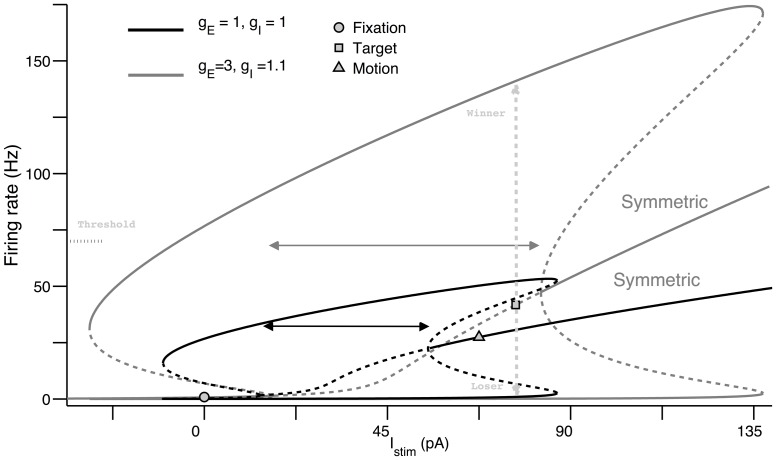
Robust decision-making regime with excitatory-inhibitory gain increase. Stability diagram of a single selective excitatory population as a function of the net stimulus input 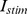 with zero motion coherence. Black: without gain modulation, 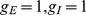. Grey: gains increase to 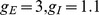. Solid and dashed lines are the stable and unstable steady-states, respectively. Double horizontal arrows show the range where a symmetric unstable steady state (dashed symmetric curves) co-exists with asymmetric stable steady states (upper and lower stable branches). These are the dynamic ranges of decision-making under these two conditions. Circle, triangle and square represent the fitted firing rate for the net stimulus input during fixation, target and motion periods, respectively. Vertical dashed double arrows show the winner-take-all effect (from the square) during motion stimulus and gain increase, either transiting to the upper winning branch or lower losing branch. Note that with gain modulation, the upper branch is mostly higher than the 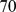 Hz response threshold, enabling saccade initiation to the winning direction.

Figure 5 plots the stability diagram as a function of net (target and motion) stimulus input 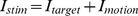 for a single selective excitatory population in the absence (black) and presence (grey) of excitatory-inhibitory gain modulation. Each of these consist of stable (bold lines) and unstable (dashed lines) loci of steady states. The upper and lower stable branches denote the ‘choice’ attractors for the winning and losing selective populations, respectively. Two of the stable loci and one unstable loci together form a continuous smooth line (highlighted with the word symmetric in Figure 5) that represents the symmetric steady states along the phase-plane diagonal in Figure 4. Its stability changes from stable to unstable and back to stable (Figure 5) as the net stimulus input increased; the unstable region (double arrowheads) is where decision-making is possible. One can already easily see that this decision-making regime with excitatory-inhibitory gain modulation (grey double arrowheads) is significantly much larger than without gain modulation (black double arrowheads); i.e. co-modulation of excitatory-inhibitory gains can lead to enhanced robustness of the decision-making process.

As in [56], in the absence of gain modulation, the selective excitatory populations transition from a low-activity spontaneous attractor during fixation epoch (circle, Figure 5) to a high-activity attractor during the choice target epoch (triangle, Figure 5) due to the choice target input, without forming any decision. Later in the trial, the motion stimulus further increases the net stimulus input. Since the symmetric curve increases monotonically with net stimulus input, this further increase in the input would force the symmetric steady state further right in the stability diagram than during the target period. Although this state has a higher firing rate, it is stable and thus does not allow any winner-take-all dynamics and decision making.

Previous modelling work solved this problem by reducing the input current due to choice targets upon motion stimulus onset [56], [69]–[71], by assuming divided covert attention from choice targets to the motion stimulus. The explicit reduction in target input compensated for the increase in input at the onset of motion stimulus. However, this manipulation leads to restrictions in the dynamical range of the neural firing rates. For example, the firing rates diverge at a level lower than the pre-motion stimulus firing activity level, contrary to some experimental findings [57], [73]. Furthermore, the choice targets remain on display throughout a trial, rendering this implementation questionable. In this work, we provide an alternative biologically plausible mechanism of modulating the gains of both excitatory and inhibitory neurons. Despite the increase in net stimulus input due to the motion stimulus, gain modulation enables a transition from the symmetric attractor during the target period (triangle in Figure 5) to a higher activity, symmetric unstable steady state during the motion period (square in Figure 5). The firing rates thereby diverge from a higher activity level during the motion period than that of the adapted target firing rate.

In addition to making the decision process more robust and more dynamic, excitatory-inhibitory modulation can also create a wider range of firing rates that can be achieved when storing categorical choice (compare the firing rates of the black and grey upper stable branches, which represent the neural storage of choice). The higher firing rates for the grey upper stable branch can more easily allow a fixed motor threshold to be crossed, i.e. motor action to be initiated. It is precisely this flexible mechanism that allows it to be also used for other behavioural task paradigm e.g. the FD task, which we will later show. It is further noted that these wide range of firing rates can also allow this threshold (currently fixed at 70 Hz) to vary more widely, adding another dimension towards more flexible decision-making strategy for speed-accuracy trade-off [68], [74]–[76], provided there is a separate independent neural mechanism to instantiate such a decision/motor threshold [77], [78].

**Figure 6:**
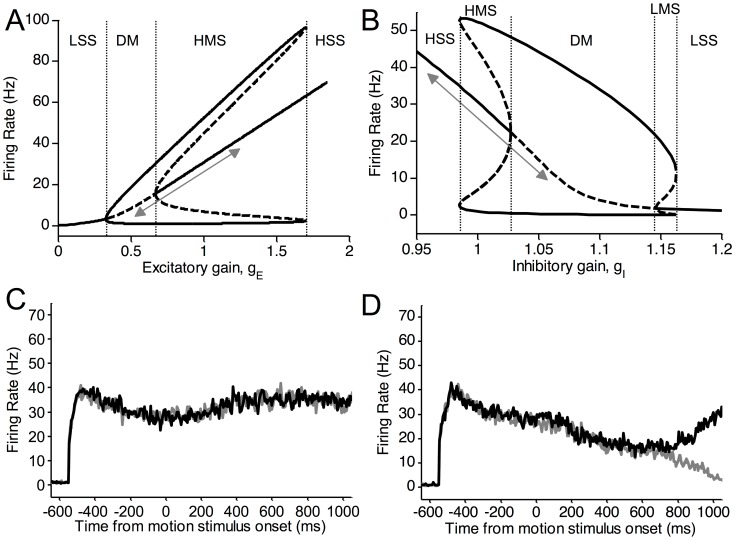
Excitatory or inhibitory gain modulation alone results in restrictive neural dynamics. (A,B) Stability diagrams of a single selective excitatory population as a function of excitatory gain 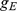 (A) and inhibitory gain 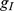 (B). Arrows in (A) and (B) show direction of change as 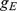 or 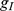 varies, respectively. Vertical dashed lines partition regimes of 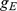 in (A) and 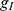 in (B), respectively. A regime can have a single symmetric stable steady state, which is either low (LSS) or high (HSS), or multiple stable steady states: one symmetric and two asymmetric, with two asymmetric unstable steady states. The symmetric steady state can be low (LMS) or high (HMS). Or it may have a symmetric unstable steady state with asymmetric stable and unstable steady states. This constitutes the decision-making (DM) regime. (C,D) Sample activity timecourses showing either no winner-take-all behaviour (C) or divergence at low firing rates, when the excitatory (inhibitory) gain is increased (decreased) in isolation (C), or when the excitatory (inhibitory) gain is decreased (increased) in isolation (D), respectively.

To further demonstrate the inflexibility of either excitatory or inhibitory gain modulation as opposed to their co-modulation, we plot the stability diagram of a single selective excitatory population with respect to each (excitatory or inhibitory) gain parameter. Figure 6A shows, for a fixed stimulus input, the effects on the excitatory neural population as the excitatory gain 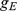 is varied with the inhibitory gain 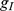 fixed at the control value of 1. As 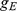 is increased from 1, the selective populations can transition from a regime with multiple high stable (HMS) steady states (including a symmetric stable state) to one with a very high single stable (HSS) firing activity. Similar transitions occur if we decrease the inhibitory gain 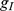 from 1 (Figure 6B). This means that increasing (decreasing) excitatory (inhibitory) gains in isolation does not allow any winner-take-all dynamics, as both populations fire at the same level (Figure 6C).

Conversely, we may decrease (increase) excitatory (inhibitory) gains in isolation. The selective populations transition through the decision-making (DM) regime (where symmetric unstable steady states coexist with asymmetric attractors) to a single low stable branch (low single steady state, LSS). Increasing the inhibitory gain leads to an additional regime with multiple steady states including a low spontaneous stable state (LMS). The decision-making regime has a lower firing rate than that during the target period. Consequently, the firing rates of the selective populations diverge at a lower firing rate than the adapted target firing rate. Thus a decrease (increase) excitatory (inhibitory) gain in isolation can enable decision-making but at a smaller dynamic range (e.g. lower firing rates) and slower decisions (Figure 6D) than found in experiments [57], [73].

**Figure 7:**
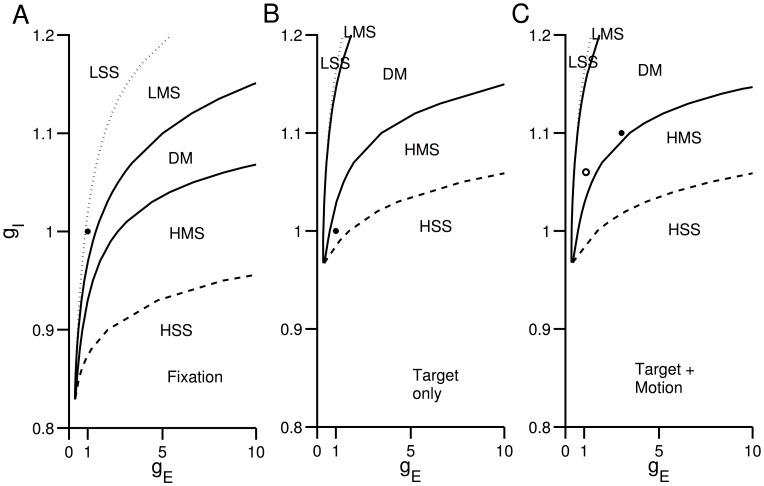
Dynamical regimes for excitatory and inhibitory gains. Distinct regimes of the network's operation as a function of excitatory and inhibitory gains 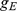 and 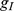, respectively. A regime can have (i) a symmetric low single stable steady state (LSS), (ii) a symmetric high single stable state (HSS), (iii) multiple stable steady states: one low symmetric and two asymmetric (LMS) with two asymmetric unstable steady states, (iv) multiple stable steady states: one high symmetric and two asymmetric (HMS) with two asymmetric unstable steady states and (v) a symmetric unstable steady state with asymmetric stable and unstable steady states, which constitutes the decision-making (DM) regime. Compare with Figure 6 A,B. The regimes of 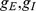 are analysed for different net-stimulus inputs (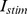), i.e. during (A) fixation, (B) target, and (C) target and motion. Black dots show our fitted 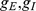 parameters during these epochs, with black and open dots showing the fitted parameters during the RT and FD tasks, respectively.

To more completely understand the interplay between stimulus inputs and gain modulation parameters, we extend the stability analysis of Figures 5 and 6A, B to the gain (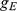, 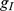) space for different epochs (and hence overall stimulus inputs) within a trial. As observed in Figure 6B (and Figure 5), in general, there are five distinct dynamical regimes for a single excitatory selective population (LSS, LMS, DM, HMS and HSS). We can see in Figure 7 that in the gain space, the range for 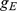 is larger than that for 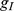 due to the generally steeper input-output function of inhibitory than excitatory neurons. More so, it should be noted that our inhibitory gain modulation is not exactly multiplicative (see *Materials and Methods*).

During the fixation epoch (Figure 7A), 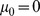 Hz, the state without gain modulation (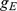, 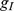)
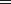
(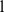, 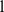) lies within the LMS regime (black filled circle, Figure 7A). We have implemented the state in this regime purely to be consistent with previous modelling work [55], [56]. However, a trial need not necessary have to start within this regime; an alternative regime could be LSS (e.g. with a smaller 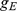 value). In the presence of a target stimulus of a (adapted to) firing rate of 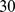 Hz, all the dynamical regimes generally shift upward (Figure 7B). In particular, the LMS regime becomes much smaller and not conducive for the population state to exist there, unless with very precise fine-tuning. It therefore makes sense for the transition to the HMS regime. Again, in principle, (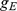, 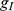) need not be (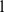, 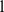) (black filled circle, Figure 7B), but can be anywhere within the HMS or even the HSS regime as long as the activity is high. We chose (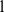, 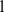) to fit the neural data in [57]. Note that the DM regime has significantly increased at this stage of the trial.

With both choice target and motion stimulus onset (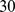 + 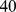 Hz, respectively), the dynamical regimes are only slightly altered (Figure 7C). This is because the overall input into each selective population is primarily dominated by the target input (Figure 2C). The DM regime is rather wide in both Figures 7B and C. The black filled circle in Figure 7C ((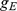, 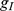)
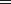
(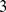, 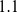)) shows the model's fit to both neural and experimental data (Roitman and Shadlen (2002)), which is close to the transition of dynamical regimes (bifurcation point) between DM and HMS. Typically when approaching such a boundary, the dynamics of the network is generally slow, and the RT distributions can exhibit long tails [72], [79]. However, if we have gains that continuously increase over time (creating a form of ‘urgency’ signal), we can curb such behaviour (Figure 3D), which is not observed in [57] (see Figure 3E; [15], [65]).

Overall, Figure 7 summarizes our analyses and shows that our model's gain parameters are robust and insensitive to small perturbations, and yet, tightly constrained by both neural and behavioural data. In particular, Figure 7 shows that for any afferent input, an increase in inhibitory gain alone can lead to more robust dynamical regimes than with only excitatory gain increase. However, the firing rate of the symmetric unstable steady state would become too large (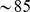 Hz, see Movie S2) and would not fit the experimentally observed divergence point of firing rates [57]. The dynamical regimes robustness can be further and continuously enhanced by following an appropriate increase in both excitation and inhibition, i.e. a larger increase in excitatory than inhibitory gains.

### Low gains in a cued response task with fixed viewing duration (FD) and a delay period

#### Comparison between model and experiment

Unlike the RT task, in a cued-to-response version of the decision task with a fixed viewing duration (FD), participants may not need to search for a trade-off between response time and accuracy, and emphasize only on the latter [57], [80]. We hypothesize that such FD tasks require lesser cognitive effort, and hence lesser (one quarter) gain modulation values in our model than in the RT tasks. Our fitted gain parameters are selected to be within the decision-making regime (Figure 7C, opened circle).

**Figure 8:**
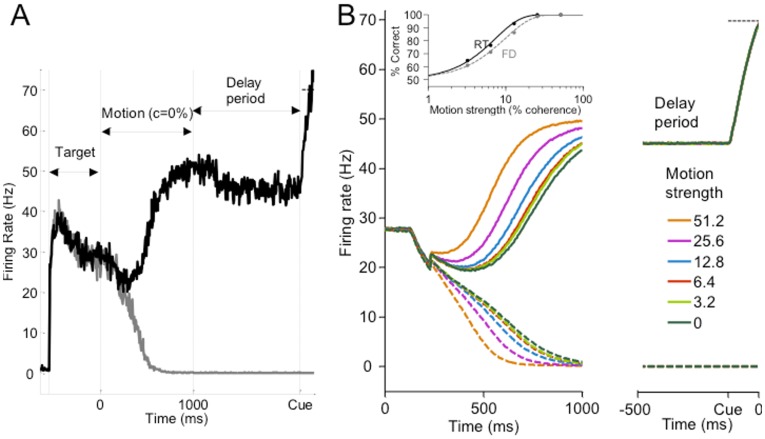
Network model reproduces neural and behavioural data in the FD task. (A) Sample activity timecourse for zero motion coherence of model with weaker gains (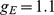 and 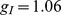). motion stimulus duration: 1 second. Delay period: 1 second. (B) Activity timecourse of model averaged across trials. Inset: Accuracy from model simulations, compared with that in RT task. Coherence threshold 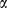 and slope 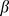 of model (experiment): 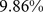 and 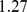 (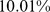 and 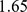), respectively. The ratio 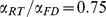 is similar to that in the experiment.

In the FD task experiments, the neural firing rates during the 1 second motion stimulus epoch are found to be generally lower in the FD task than in the RT task (Figure 1C,D). Furthermore, the divergence of firing rates are also lower in the FD task. In fact, this divergence can even take place at lower activity level than the (adapted) target firing rate [18], [80]. Most importantly, the firing rates in the FD task are observed to diverge during motion presentation but maintained at a low activity level during the delay period. Our model with low gain modulations can account for these neuronal effects (Figure 8A, B) of [57]. The lower gains cause slower ramping of neuronal activity (compare with Figure 3A). Although the fixed viewing duration of 1 sec would allow sufficient time to integrate sensory information with higher gains, for lower gains it does not. Thus, decisions are less accurate in the FD task than in the RT task (Figure 8B inset). This is also consistent with the account in [59].

In Figures 8 A and B, we can see that during the motion stimulus period, although the firing rates have already diverged and thus the decisions formed, the activities are maintained at levels lower than the response threshold of 70 Hz. This is achieved by having lower excitatory and inhibitory gains. Thus, in the FD task, it is clear that the threshold is actually a motor response threshold rather than a decision threshold per se; the decision is already formed during motion stimulus presentation prior to the delay period and response cue. When the cue to respond is presented, the gains are increased to values as in the RT task. The firing rate of the winning population rapidly crosses the threshold and a corresponding saccade is initiated.

### Strong recurrent excitation is not necessary for forming and storing decisions

**Figure 9:**
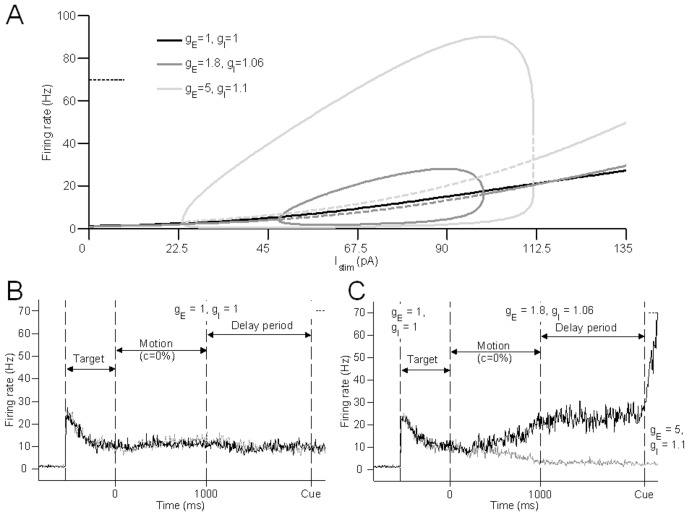
Weak recurrent excitation in the FD task. (A) Stability diagram for a single selective excitatory population as a function of net stimulus input current 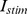, with weak recurrent excitation (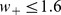) in the absence of gain modulation (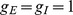), with low gains (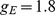, 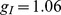) and large gains (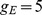, 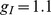). Solid and dashed lines show stable and unstable steady states, respectively. (B) Without any gain modulation, the network cannot perform decision-making nor store decisions. (C) With sufficient gain modulation, network can form a decision and store it below a motor threshold (dotted horizontal line at 70 Hz). Upon cue to respond, higher set of gains allow threshold to be crossed and saccade to be made.

Previous work has suggested that strong recurrent excitation is necessary for the formation of a decision and its maintainence in working memory during a delay period [55], [59]. In the absence of gain modulation, with weak recurrent excitation (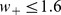), the stability diagram with respect to the net stimulus input current is shown in Figure 9A (black trace). Since there is neither any unstable steady state nor choice attractors, the network is incapable of forming or maintaining decisions (Figure 9B). However, with excitatory and inhibitory gain enhancements, for the same weak recurrent excitation and range of net stimulus input current, the network can have a symmetric unstable steady state (Figure 9A, dark grey trace) and can still perform very similar functions of decision formation and storage (Figure 9C) as in Figure 8A. We have used a higher excitatory gain 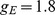 compared to the 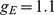 in Figure 8A, while maintaining 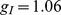 in both cases.

Although there is no intrinsic hysteresis in its stability diagram (i.e. no LMS), the network can still sustain its decision formed throughout the delay period (with the removal of motion stimulus input) as long as the target stimulus remains (which is the case in [57]). Upon response cue onset, the excitatory and inhibitory gains are increased (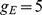, 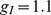), so that the upper stable branch, i.e. of the winning ‘choice’ attractor has firing rates that are higher than the motor decision threshold (Figure 9A, light grey trace) enabling the crossing of the saccadic threshold (Figure 9C).

Thus, we have demonstrated that with co-modulation of excitation and inhibition, strong intrinsic recurrent excitation is not necessary for decision formation and storage. This can be explained heuristically by first noting that the input due to recurrent excitation 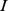 is proportional to 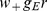, where 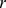 is the (pre-synaptic) firing rate. Due to the multiplicative nature of these parameters and variables, a high excitatory gain 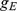 can compensate weak recurrent excitation 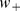.

### Optimal decision performance with fast recruitment of gains

We have so far been assuming a single time constant of gain modulation. However, this fitted time constant may not necessary be the optimal time constant for the tasks which we have discussed. In particular, a RT task involves a speed-accuracy tradeoff: slow RTs are more accurate while fast RTs may lead to more errors [54],[81]. Since only the correct trials are rewarded and error trials are penalised with a lengthened trial duration, performance can be measured by the average reward rate, which can be quantitatively defined as the total number of correct trials divided by the total time duration spent in a block or multiple blocks of trials. Thus, a form of optimal performance in RT task would require maximizing the reward rate. The time duration of a trial not only depends on the subject's RT but also on the experimental task design (e.g. inter-trial interval, and various other temporal delays). In particular, a trial in [57] can consist of several temporal delays, each contributing to the trial duration [15]: (i) between appearance of the fixation point and monkey achieving stable fixation; (ii) before the appearance of choice targets; (iii) before motion stimulus was presented; (iv) the recorded RT of the monkey; (v) saccade duration; (vi) a possible delay before reward was provided depending on whether the choice was correct; and finally, (vii) an inter-trial interval before the reappearance of the fixation point. When calculating the overall time spent within a trial, we follow the procedure in [15]. The average trial duration (TD) is as follows.

On correct trials:
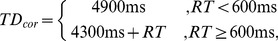
(6)which accounts for the fact that the subjects had to wait a minimum time after the onset of the motion stimulus before which reward was delivered.

On error trials:
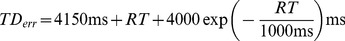
(7)which takes into account the timeout following error choices and the RT dependent timeout that was imposed to prevent impulsive guesses. For simplicity, we do not include trials on which fixation was broken without an immediate saccade being made to a choice target. These were rare in the experiment of [57] and not included in the data set used in their publication (Jamie Roitman, personal communication).

The mean reward rate for a trial of a particular coherence is given by
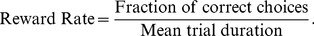
(8)


The mean trial duration for a particular coherence is the weighted mean of the average trial durations for correct and error trials. Since all six coherences used were uniformly distributed within each block, the overall fraction of correct choices was simply the arithmetic mean of the fraction of correct choices for the individual coherences. Similarly, the overall mean TD was the mean of the mean trial durations for the six coherences.

**Figure 10:**
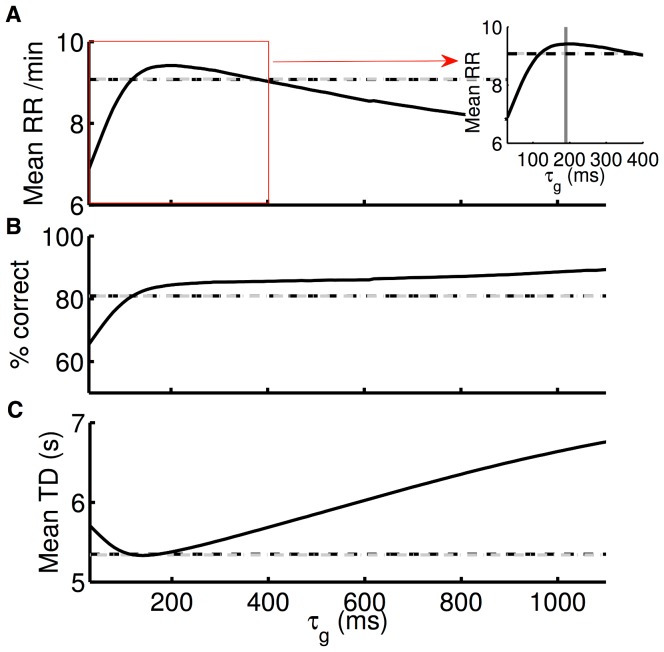
Optimal timescale of gain modulation for maximizing mean reward rate. Network model performance as a function of the time constant of gain modulation 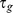. (A) mean reward rate (RR); (B) accuracy; (C) mean trial duration (TD). Dashed horizontal lines show our model's fit to the data, with 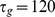 ms. Inset: zoomed in around the optimal timescale, showing the optimal timescale is 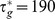.

In order to understand how fast gain modulation should be recruited in an RT task, we calculated the overall mean reward rate as a function of the time constant of gain modulation 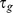 (Figure 10A). We find that the optimal time constant of gain modulation operates at a relatively short timescale with 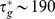 ms (Figure 10A inset), for which accuracy is maximized while the mean TD is minimized (Figure 10B, C, respectively). This short time constant indicates the need for the fast recruitment of gain modulation in order to maximise reward rate. Our fitted time constant of 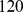 ms suggests that although monkeys in the experiments are not performing optimally, they are not too far from optimality. In particular, recruiting gain with a time constant of 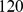 ms would cost an average reward rate of 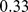 rewards per minute.

**Figure 11:**
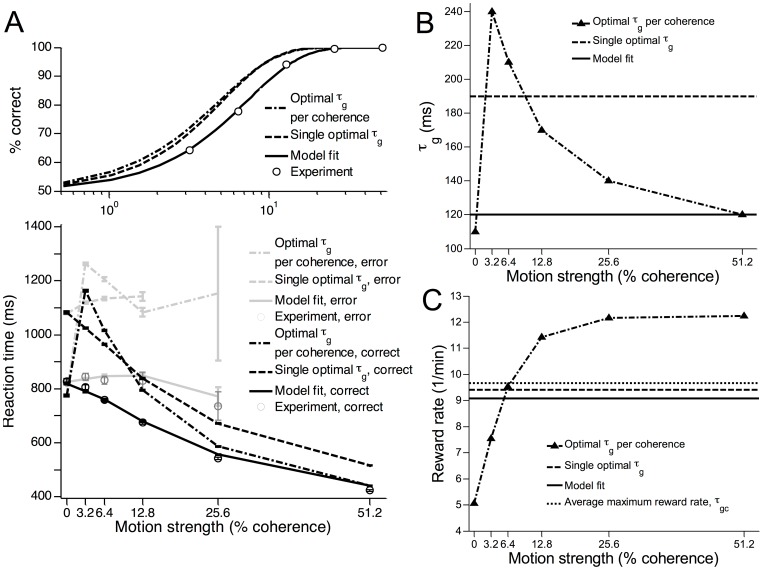
Optimal timescale of gain modulation for maximizing reward rate for individual coherences. (A) Network's accuracy (upper panel) and RT (lower panel) with the optimal timescale of 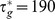 ms (dashed), compared with experimental data (open circles), and our previous model's fit to it with 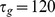 ms (black bold). Dash-dotted: model with an optimal gain time constant 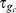 for each motion coherence. Lighter colours in the lower panel are for error RTs. Error bars denote standard errors of the mean. (B) Comparing optimal 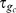 for each individual motion coherence (triangles, dash-dotted curve) with model fit (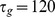 ms, (black bold line)), and single optimal (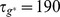 ms, (dashed line)). (C) Comparing RR for optimal 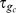 for each individual coherence with that of model fit (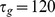 ms), and single optimal (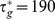 ms). Dotted: average of the RR with 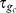 over all motion coherences.

Modelling the behavioural data with this optimal time constant produces left-shifted psychometric curves with a coherence threshold 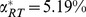 and a slope 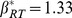, corresponding to a better performance than the experimental data and our model's fit to it (Figure 11A, upper panel). On the other hand, chronometric curves are shifted upward, revealing slower responses when compared to the experiment and our model's fit (Fig. 11A, lower panel), a consequence of the speed-accuracy tradeoff.

When calculating this optimal gain time constant, we have assumed that subjects recruit gain modulation with the same timescale throughout multiple blocks of trials, irrespective of task difficulty (motion coherence). However, if coherences are known, higher reward rates may more likely be achieved by the optimal recruitment of gain modulation for each individual coherence [82]. This could be possible for experiments in which the coherence is fixed within a block. Then the optimal gain time constant decreases with coherence, except for zero motion coherence, where it is near our model's fit of 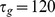 ms (Figure 11B). For lower non-zero motion coherences, it is longer than the single optimal gain time constant, but shorter than that for higher ones. Since the optimal gain time constants are larger for lower coherences (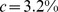 and 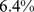), a longer duration of evidence accumulation becomes possible, and thus, RTs are lengthened (Figure 11A, upper panel) and accuracies are improved (Figure 11A, lower panel).

This task-difficulty dependent gain modulation strategy has a reward rate (averaged over the motion coherences) that is higher than that of the previous task-difficulty independent gain modulation strategy. In fact, this difficulty-dependent strategy can lead to an average reward rate of 9.66 rewards per minute, which is 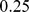 rewards per minute greater than the reward rate using a single time constant of gain modulation (Figure 11C) throughout multiple blocks of trials.

The optimal time constants are applicable when we use the realistic temporal delays of [57]. These impose temporal penalty delays to discourage the monkeys from responding impulsively. Eq. (7) shows that fast, erroneous responses elongate the error trial duration (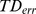) more than slow ones according to the 
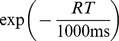
 term (see the dashed curves in Figure S3A, lower panel). Although theoretically, an erroneous response on a 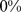 coherence condition is ill-defined, responses for this condition in the experiment were randomly (with probability 0.5) deemed erroneous (see Figure S3A, middle panel) and thus also incurred the error delays. Since accuracy on a 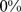 coherence condition is 0.5, thus 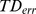 and 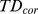 are equi-probable. The reward rate (see Eq. (8)) for responses that are too fast is therefore lower (see Figure S3A, upper panel). Furthermore, the minimum trial duration (see Eq. (6)) even for correct responses also discourages fast responding. However, if only RTs, plus a fixed inter-trial interval (Figure S3 B, lower panel) instead of 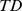 were used to maximize reward rate [81], then the optimal time constant for a 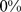 coherence would indeed approach 0, i.e., it would be optimal to respond randomly and immediately (Figure S3B, upper panel).

## Discussion

Top-down cognitive control such as attention has been suggested to form an integral component in perceptual decision-making [30]. Our current study is inspired by the findings in [52], which show that attention can induce gain modulation of both excitatory and inhibitory neurons, and also by those of [53], which suggest that attention can have a time-varying nature. However, there has not yet been any study on how time-dependent gain modulation of both excitatory and inhibitory neurons can affect decision dynamics and performance.

Specifically focusing on the two highly-studied reaction time (RT) and fixed delay (FD) decision task paradigms, we use both computational simulations and dynamical systems analysis of a biologically inspired decision-making model to address this issue. In our study, we have shown that simultaneous dynamic gain modulation of both excitatory and inhibitory neurons is capable of reproducing the experimentally observed dynamic range of neural activities throughout an entire trial. Our model is able to robustly reproduce realistic temporal dynamics including the signature dip phenomenon in the firing rates (shortly after motion stimulus onset) without artificially lowering the overall stimulus input as implemented in previous modelling work [56], [69], [70], [71]. Interestingly, there is some evidence to show that this dip is possibly related to lateral inhibition in the neural circuit [83]. We are also able to replicate the behavioural data of the monkey experiments, including slow mean and short-tailed RT distributions even when the network is operating near a dynamical bifurcation point (Figures 5 and 7C).

Without specifying any particular neural mechanism, our excitatory-inhibitory gain modulation can allow the same local cortical circuit to flexibly adapt over time to different decision-making task demands. By adopting higher gains in the RT than FD task, we were able to capture not only the behavioural data as in previous models [55], [56], [59] but also better replicate the neuronal activity timecourse of recorded LIP neurons. In particular, our model suggests that the presumed decision threshold in the FD task could actually be more of a motor activation threshold rather than an actual decision threshold – the decision is already made during the stimulus presentation. Our weaker gain implementation is based on the hypothesis that a RT task, which requires optimizing a speed-accuracy trade-off, is more cognitively demanding than the FD task.

When both excitatory and inhibitory neuronal gains are co-modulated, our model becomes more robust to small changes with respect to the stimulus input (Figure 5). Furthermore, Figure 7 shows that decision-making computations are robust when both gains are increased by an appropriate amount. From a broader perspective, this adds further support to our previous work that modulation of both excitation and inhibition is necessary to produce robust decision-making without sacrificing optimal decision performance [72], [84]. In a more realistic setting, decision-making is usually influenced by a multitude of (e.g. sensory) information through modulation of the neuronal firing rate during temporal integration [56], [73]. Thus, it may be important to have a decision network operating with a larger capacity to allow more potentially useful information to be stored during sensory integration. Such higher information storage capacity or larger decision bandwidth has recently been investigated in other contexts [71], [85]. This decision bandwidth was previously shown to be relatively small with weak recurrent excitation (small 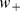) [55].

In the absence of gain modulation, weak recurrent excitation can lead to decisions made not being stored in working memory (owing to an inability to sustain neural firing activity), and can prevent the subsequent motor action from being triggered (owing to neural firing activity which is lower than the motor threshold) [55]. With even weaker recurrent excitation, a decision may not even be formed at all [55]. This could impede performance in the FD task, which requires working memory during a delay period [84]. Our work here demonstrates that enhanced excitatory and inhibitory gains can compensate such weak recurrent excitation to make decisions, and even store them in working memory.

Our gain modulation mechanism is thus more flexible than conventional decision-making models [55], [56], [59]. Incidentally, Figure 9C is comparable to experimental data in [18], [80]. Recent experimental evidence has shown that the parietal cortical neurons without persistent activity can still show some form of decision-making (winner-take-all) capabilities [86], thus supporting our proposed mechanism (see Figure 9A). Furthermore, by reducing the gains in our model after threshold crossing, we can also easily clear any storage of decisions, resetting the system towards a low single stable state (LSS) at the end of a task trial (Figure S2), as observed in many experiments [17], [57], [73], [80]. This deviates from previous computational work, which require negative current [87] or transient synchronized firing [88] to reset the system. Clearly, to inform such post-decision shutting down of gains, a different neural circuit may be necessary, and the basal ganglia may be a putative candidate [77], [89].

Previous decision-making models have also incorporated time-varying gain modulations or urgency signals to allow flexible reconfiguration in the model dynamics, or in some cases, attempted to capture the characteristics of (especially LIP) neuronal firing rates throughout a decision-making trial [6], [13]–[17], [21], [54]–[56], [65], [69]–[71], [82], [89], [90]. Although the models that incorporated urgency signals [15], [16], [21], [54] share some similarities to our model, the instantiations of these models differ in distinctive ways. Specifically, in [15], the urgency signal multiplies the instantaneous evidence (i.e. drift rate and noise in a drift-diffusion process), while in [21] the decision bound or threshold generally decreases over time. In a two-choice task, these two mechanisms are equivalent [16]. The work in [54] resembles our model the most, in which the slope of the input-output function of the population firing rate is modulated over time and the decision network transits from a leaky to a competitive integrator (see also similar discussions in [91], [92]), whereas our gain modulation mechanism is more multiplicative (see Eq.(1)). Generally, these models are not as biologically grounded as our model, which was previously reduced from a spiking neuronal network model [55], [56]. Our more realistic model is able to directly compare and qualitatively account for the full dynamical range of the LIP neuronal activity throughout a trial (e.g. compare with [57], [73], [80]). In addition, as in [15], our model uses realistic temporal delays and replicates the reaction time distributions in [57]. Finally, the most important distinctive feature in our model is that it incorporates the gain modulation of the inhibitory neural population for flexible and robust decision-making, which none of the previous modelling work has investigated.

Our work also demonstrates, for the first time, how dynamic excitatory-inhibitory gain modulation in a biologically realistic model can give rise to optimal decision performance. Using realistic temporal delays from the experiment of [57], we found that our model's excitatory-inhibitory gain modulation timescale (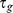) that maximizes reward rate (RR) in a RT task is not far from the value we obtained when fitting both the neural and behavioural data. This suggests that the monkeys may be performing not far from optimality, although a more in-depth study similar to [93] may be required to further support this claim. Interestingly, we found that the fitted gain time constant resides slightly on the left and steeper side of the RR-vs-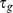 (Figure 10A) curve, i.e. 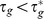. It would have been less cognitively demanding for the subject to operate around the shallower right side of the curve, where the cost of RR is lesser. The optimal gain time constant found in our model may not be the true value, but simply the optimal value given all other parameter settings. Precise experimental verification will be required to measure and disentangle the contributions of different parameters. Alternative model parameter settings such as with a higher noise level or a different MT neuronal firing output (e.g. weaker dependence on motion coherence in the anti-preferred direction) would lead to different optimal gain time constants (Figures S4, S5). However, changing these would introduce additional free model parameters. In this work. we prefer to maintain the model parameters based on previous work, especially since the main focus of our work concerns the flexibility and robustness afforded by the co-modulation of excitatory and inhibitory gains. It is however interesting to note that the fast 120 ms time constant we used to fit the data is similar to that of the urgency signal deduced in [21] from the same dataset [57].

The first part of our optimal performance study is based on the assumption that subjects employ the same ‘strategy’, and thus a fixed timescale of gain modulation throughout the whole block of trials. However, in experiments in which coherence is fixed within a block [76], [81] and can be determined, adjustment of gains based on task difficulty is the optimal strategy in the RT task. We confirm this in the second part of our optimal reward study, which shows that by strategizing the timecourse of gain recruitment (e.g. via rapid feedback) for different task difficulty (e.g. motion coherence), a higher optimal RR can be achieved. The main contribution for higher RR, comes from the higher motion coherences (easier tasks). The task-difficulty dependent recruitment of gains, however, may not be practically adopted by subjects. It is plausible that this flexible, more optimal strategy may be too demanding to be practically implemented. The slight increase in RR may not be worth the cognitive effort ([76] make a similar argument in the context of response threshold modulation instead of gain modulation). Furthermore, our optimal time constants are applicable when temporal delays such as those in [15], [57] are used to penalise fast errors more than slower ones. Since in this case, fast errors reduce the reward rate more, the optimal time constant of gain-modulation gets skewed towards a longer time. Not including such temporal penalisations, but using a fixed inter-trial interval leads to much shorter optimal time constants of gain modulation (see Figure S3B upper panel). Assuming that the subject knows the coherence, the optimal time constant for the 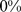 coherence condition then approaches 0, since the subject can receive more trials and increase its reward rate by guessing as fast as possible. Provided subjects are attempting to maximize reward rate, the optimal time constant should be much shorter on tasks that include a fixed inter-trial interval (e.g. [81]) and longer on those which penalise (either in time or through explicit punishments) fast errors.

Finally, it would be interesting to test our proposed simultaneous excitatory-inhibitory gain modulation mechanism in behaving animals performing perceptual decision-making tasks. For example, the task can include a fraction of trials with some cue to capture the subject's attention, while putative excitatory and inhibitory neurons are recorded. The neural activities and behavioural performance could then be compared between trials with or without the attentional cue.

## Materials and Methods

Following [55] and [56], the non-linear input-output function of a single noisy excitatory cell is approximated from the first-passage time input-output relation of a leaky integrate-and-fire neuron [46], [55], [60].
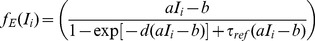
(9)where 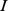 is the total synaptic input current to a neuron, and 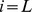 or 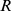, denoting selectivity to a leftward or rightward motion stimulus, respectively. We follow the fitted parameters of 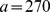 Hz/nA, 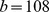 Hz and 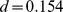 s as in [55]. 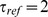 ms is the absolute refractory period of the neuron, although our results are similar if we ignore this term.

For the inhibitory interneuronal population, we assume that 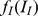 is linear so that it can be implicitly embedded in the reduced two-variable model for analysis [55]. This yields 
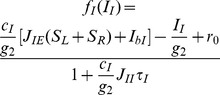
, where 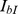 is the background input current to the interneurons, 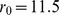 Hz, 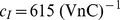, 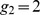 and 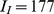 Hz are parameters from the first-passage time input-output relation of a leaky integrate-and-fire interneuron. The decay time constant of GABA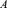 receptors is relatively much faster (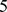 ms), and so the inhibitory gating variable 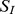 can be assumed to quickly achieve steady state, i.e. 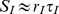.

We can now reduce our model into an effectively two variable one by implicitly embedding the inhibitory coupling 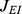 in 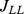, 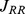, 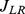 and 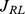 and 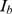 of Eq. (2), such that
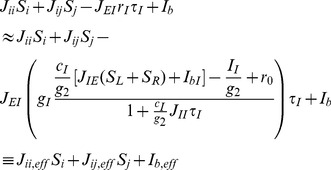
(10)with
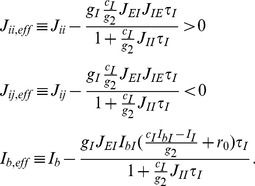
(11)Note that 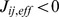 is required to allow competition via effective mutual inhibition between the two selective excitatory populations. This means that 
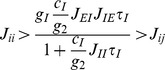
. To allow all defined 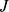
's to be 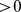, we shall replace 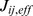 with 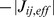. To simplify the notation, we shall henceforth remove the label “eff”. Note that this effectively makes our implementation of inhibitory gain strictly non-multiplicative, although the results would be similar if it was; in fact, multiplicative gain modulation is sufficient but not necessary for producing our results [91].


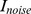
 in Eq. (2) is assumed to be primarily filtered by fast AMPA receptors (with decay time constant 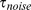 of 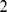 ms) via an Ornstein-Uhlenbeck process [55], [94]

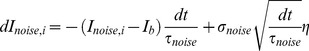
(12)where 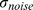 is the standard deviation of the noise and 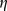 is Gaussian distributed white noise with zero mean and unit variance.

### Simulations and stability analyses

We performed noisy simulations of our model using MATLAB using a forward Euler-Marayama numerical scheme [95] with a time-step of 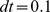 ms. Smaller time steps do not affect our results. In order to compute the average firing rates and behavioural statistics, we performed 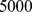 trials of noisy simulation for each set of model parameters.

The activity timecourse of our model can be best understood by analysing its dynamics on the two dimensional state/phase space called the phase plane (see Figure S1). We set both dynamical equations (Eq. (5)) to be 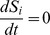 and solve for the 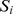
's, where 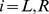. The solutions for each equation (called a nullcline) can be plotted in the (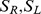) phase plane. Since the firing rates 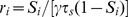 are monotonic functions of the average gating variables 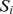, transforming to 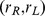 coordinates yields the same qualitative dynamics.

Intersections of the nullclines give the steady states (fixed points) of the network. For our purposes, these steady states can be stable, i.e. point attractors (Figure S1A) or (semi-) unstable, i.e. saddle points (Figure S1B). In a noiseless system, trajectories near an attractor will move towards it (with local velocities called vector fields shown by the length of the arrows in Figure S1A). For an unstable steady state, only trajectories starting on a unique curve are attracted into it. This curve is called the stable manifold of the unstable steady state. Trajectories on all other parts of the plane are repelled away from it to another associated curve, called its unstable manifold [55], [96].

The loci of all steady states (stable and unstable) as a function of a parameter yields a stability (bifurcation) diagram. Phase-plane and stability (bifurcation) analyses of our two-variable network model were done using XPPAUT [97].

### Constraining model parameters

Apart from the new parameters mentioned in Table 2, we maintained the parameters as in previous work, as reported in Table 1. These parameters were used to provide a qualitative rather than quantitative fit to the neural and behavioural data, and we simulated predictions at a range of parameter values until the desired fits were isolated. In order to constrain our new parameters, we first ensured that our excitatory and inhibitory gain maximal amplitude parameters (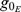 and 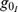) were in the dynamical regime that allowed decision-making (Figure 7). We then fitted the excitatory and inhibitory gain onset times 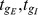 to replicate the characteristic dip in neural firing-rates at motion onset. Finally, we fit the gain modulation time constant 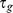 to the behavioural (reaction time and accuracy) data, while ensuring that the neural activities were not unrealistically low.

## Supporting Information

Figure S1
**Two dimensional state/phase space called phase plane.** The firing rate 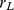 of the population selective towards leftwards (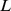) motion is plotted against the firing rate 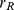 of the population selective towards rightward (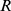) motion. Orange and green curves represent nullclines: where 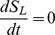 and 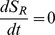, respectively. The synaptic gating variable 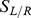 activities are transformed to firing rates, preserving the same qualitative dynamics. Intersections of the nullclines yield the steady states of the system. (A) A symmetric **stable steady state** (symmetric **attractor**). Trajectories starting near this steady state are attracted into it, with local velocities given by the arrows. This set of arrows is called the **vector field**. The set of all starting points for trajectories attracted into this attractor is called its **basin of attraction**. In this figure this is the entire phase plane. (B) A symmetric **unstable steady state**, called a (symmetric) **saddle point**. Only trajectories starting on a unique curve (shown in light blue) are attracted into it. This curve is called the **stable manifold** of the unstable steady state. Trajectories on all other parts of the plane are eventually repelled away from the unstable steady state, to another curve (shown in yellow), called its **unstable manifold**. There are also two **asymmetric attractors**. The stable manifold of the unstable steady state separates the basins of attraction of these two attractors.(TIF)

Figure S2
**Post-decision shutdown.** After the firing rate of one of the selective populations has crossed the motor threshold (70 Hz) for saccade initiation, the gains of both excitatory and inhibitory neurons are allowed to decay towards 0. (A) Phase plane at the end of a trial. The firing rate 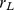 of the population selective towards leftwards (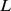) motion is plotted against the firing rate 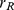 of the population selective towards rightward (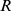) motion. Orange and green curves represent nullclines: where 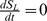 and 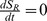, respectively Only a low, symmetric attractor is present. Post-decision, trajectories start from either the upper left or the bottom right, and move towards this attractor, with local velocities shown by the arrows (B) As a result, the firing rates of both winning and losing populations are reset to baseline, before the start of the next trial, as observed in experiments.(TIF)

Figure S3
**Task dependent optimal timescale of gain modulation for maximizing reward rate for individual coherences.** (A) using the realistic temporal delays from Roitman and Shadlen (2002). (B) in a reaction time task with a fixed inter-trial interval (ITI). Upper, middle and lower panels show reward rate (RR), accuracy and trial duration (A) or 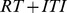 (B). The ITI shown here is 1500 ms. Solid and dashed curves in lower panels show correct and error trials, respectively. Black arrows in upper panels show how the optimal time constant of gain modulation changes with increasing coherence.(TIF)

Figure S4
**Shallower slopes in relation to motion coherence in the anti-preferred direction leads to slightly longer reaction times and poorer accuracy.** (A) Timecourse of input currents for equal slopes (black) and 2 times shallower slopes (red) of input current out of the response field, in relation to motion coherence. (B) Activity timecourse of model, averaged over multiple trials, with different motion coherences for (left) equal slopes and (right) 2 times shallower slope of input current in relation to motion coherence in the anti-preferred direction. Response threshold at 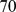 Hz, compare with Figure 1. (C,D) Accuracy (C) and mean RT (D) generated by model and in the experiment of [57]. The model with equal slopes (see also Figure 3) and a modified version with a 2 times shallower slope of input current in relation to motion coherence in the anti-preferred direction (red) are shown. (E) Network model performance as a function of the time constant of gain modulation 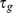 using the equal slopes (black) as in the main manuscript (see Figure 10) and another with a 2 times shallower slope (blue) of input current in relation to motion coherence in the anti-preferred direction. Upper panel: mean reward rate (RR); middle panel: accuracy; lower panel: mean trial duration (TD). Dashed horizontal lines show our model's fit to the data with equal slopes and 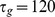 ms. Vertical lines in the upper panel show the optimal timescale of gain modulation. Inset: mean reward rate zoomed in around the optimal timescale, showing the optimal timescale is only around 20 ms shorter 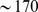
ms (compared to 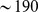
ms) when using shallower slopes for input current in relation to motion coherence in the anti-preferred direction. Shallower slopes in relation to motion coherence in the anti-preferred direction lead to a lesser discriminatory ability for the network. Thus, the firing rates for the different motion coherences ramp up/down closer together (B), resulting in poorer accuracy (C). Furthermore, the changes are more pronounced for the higher motion coherences (A) – the input currents to the losing population are generally greater, leading to greater competition between the two competing populations. This subsequently slows the integration time of the winning population, and hence lengthens the reaction time (D). This leads to a very slightly shorter optimal gain time constant (E).(TIF)

Figure S5
**Optimal timescale of gain modulation for maximizing mean reward rate for different noise levels.** Network model performance as a function of the time constant of gain modulation 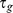 using the noise level (black) in the main manuscript (see Figure 10) and another with a higher (twice the standard deviation) noise level (red). Upper panel: mean reward rate (RR); middle panel: accuracy; lower panel: mean trial duration (TD). Dashed horizontal lines show our model's fit to the data with 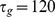 ms and noise as reported in the main manuscript. Vertical lines in the upper panel show the optimal timescale of gain modulation. Inset: mean reward rate zoomed in around the optimal timescale, showing the optimal timescale is around 200 ms longer 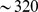
ms (compared to 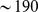
ms) for a higher (twice the standard deviation) noise level.(TIF)

Movie S1
**Dynamics of decision-making over an entire trial.** View slideshow to play. Upper and lower panels show phase-planes and activity timecourses, respectively, throughout the various epochs of a trial for coherence 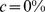. The firing rate 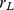 of the population selective towards leftwards (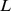) motion is plotted against the firing rate 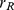 of the population selective towards rightward (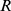) motion. Orange and green curves represent nullclines: where 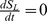 and 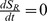, respectively. The intersection of nullclines yield the steady states. Arrows denote the local velocities of trajectories. The network starts from a low symmetric attractor during the fixation period. A burst of input current at the onset of choice targets reconfigures the network such that only a single stable steady state is present, preventing decision-making. After adaptation, the network settles to a high symmetric attractor, with additional asymmetric attractors also present. At motion onset, the inhibitory gain is increased, forming an nearby unstable steady state with a lower firing rate. The network moves towards this steady state along the diagonal line (with equal firing rates), but before it can reach it and firing rates diverge, the excitatory gain is increased, raising this unstable steady state. This reproduces the “dip” phenomenon. When the input due to the motion stimulus comes into effect, the network is reconfigured initially to a symmetric stable steady state (which prevents early divergence of firing rates), and then to a symmetric unstable steady state with a firing rate that is higher than the adapted target firing rate. This unstable steady state becomes more unstable (nullclines come closer together) as both gains increase dynamically. Consequently, the firing rates of the competing selective populations diverge and ramp up (down) more and more quickly towards the winning (losing) ‘choice’ attractor. The motor/response threshold of 70 Hz is crossed prior to the network reaching the corresponding ‘choice’ attractor, and a saccade is initiated. The increasing instability caused by both attractor network and increasing gain dynamics creates an urgency signal, leading to short tailed RT distributions, even if the network operates close to a dynamic transition (bifurcation) point.(PPTX)

Movie S2
**Larger dynamic ranges allow robust decision-making for co-modulation of excitatory and inhibitory gains.** Stability diagrams are shown as a function of excitatory gain 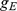 for different inhibitory gains 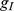. Black dashed lines show parameters that fit the behavioral and neural experimental data, namely 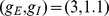. For each stability diagram as a function of 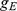, dark shades show stable branches, while light shades show unstable ones. As the inhibitory gain 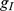 is reduced, the dynamic range of the network decreases. Furthermore, our fitted parameters are not sensitive to small perturbations. If we increase or decrease 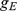 or 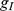 slightly, our model adequately performs its decision-making computations. However, if we had chosen a much smaller value of 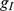 as our parameter, small perturbations in parameter values would have rendered the network incapable of performing its decision-making computations. On the other hand, we may increase 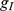, which would lead to the network performing its decision-making computations with a larger dynamic range. However, the unstable branch would then have a very large firing rate (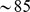 Hz) and not fit the neural experimental data.(AVI)
