# Supplementary figures and images for: Dynamic Excitatory and Inhibitory Gain Modulation Can Produce Flexible, Robust and Optimal Decision-making
Source: PLoS Comput Biol. 2013 Jun 27;9(6):e1003099. doi: 10.1371/journal.pcbi.1003099 (PMC3694816; doi:10.1371/journal.pcbi.1003099)

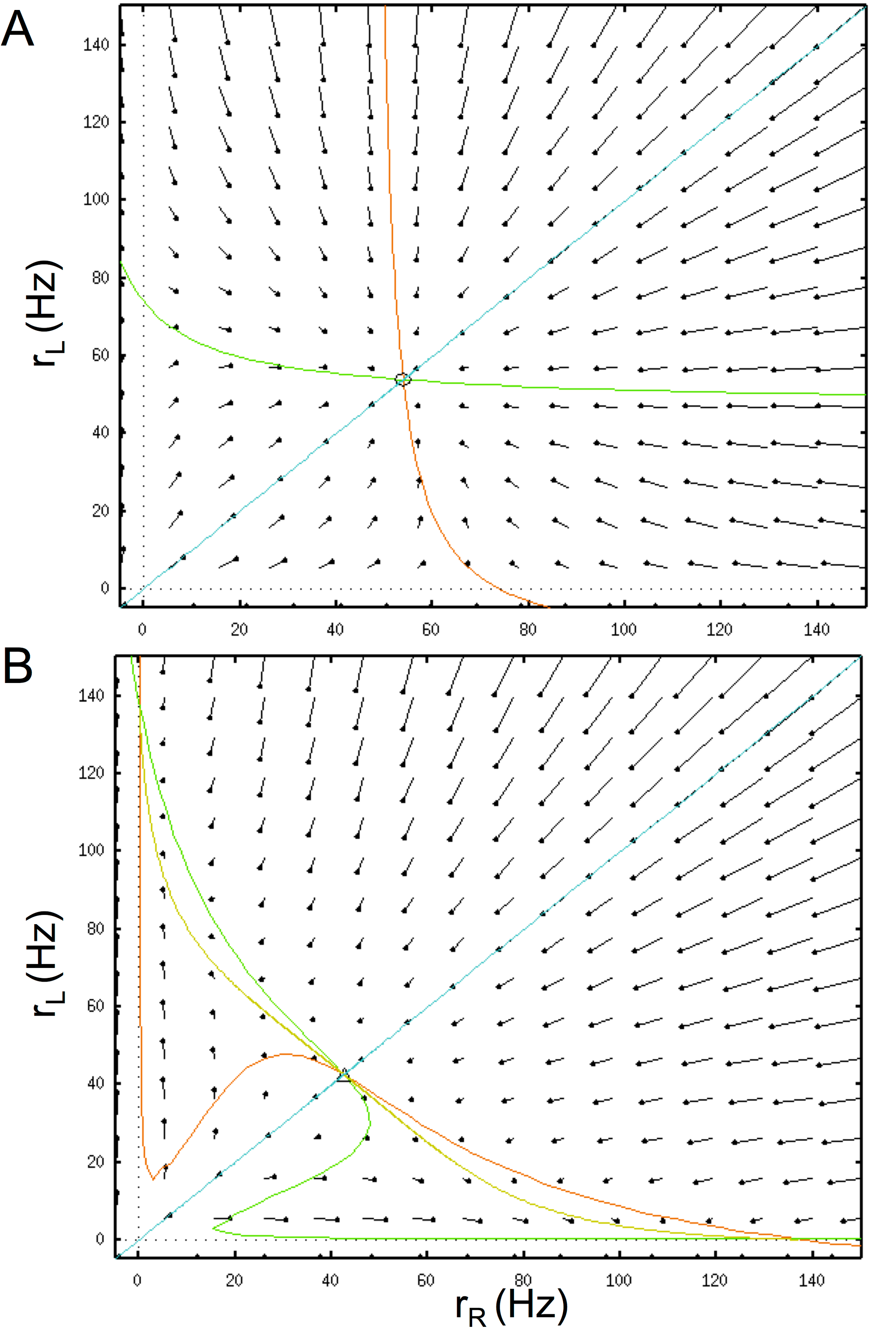

Supplement: Figure S1 — Two dimensional state/phase space called phase plane. The firing rate of the population selective towards leftwards () motion is plotted against the firing rate of the population selective towards rightward () motion. Orange and green curves represent nullclines: where and , respectively. The synaptic gating variable activities are transformed to firing rates, preserving the same qualitative dynamics. Intersections of the nullclines yield the steady states of the system. (A) A symmetric stable steady state (symmetric attractor). Trajectories starting near this steady state are attracted into it, with local velocities given by the arrows. This set of arrows is called the vector field. The set of all starting points for trajectories attracted into this attractor is called its basin of attraction. In this figure this is the entire phase plane. (B) A symmetric unstable steady state, called a (symmetric) saddle point. Only trajectories starting on a unique curve (shown in light blue) are attracted into it. This curve is called the stable manifold of the unstable steady state. Trajectories on all other parts of the plane are eventually repelled away from the unstable steady state, to another curve (shown in yellow), called its unstable manifold. There are also two asymmetric attractors. The stable manifold of the unstable steady state separates the basins of attraction of these two attractors. (TIF) [file pcbi.1003099.s001.tif]

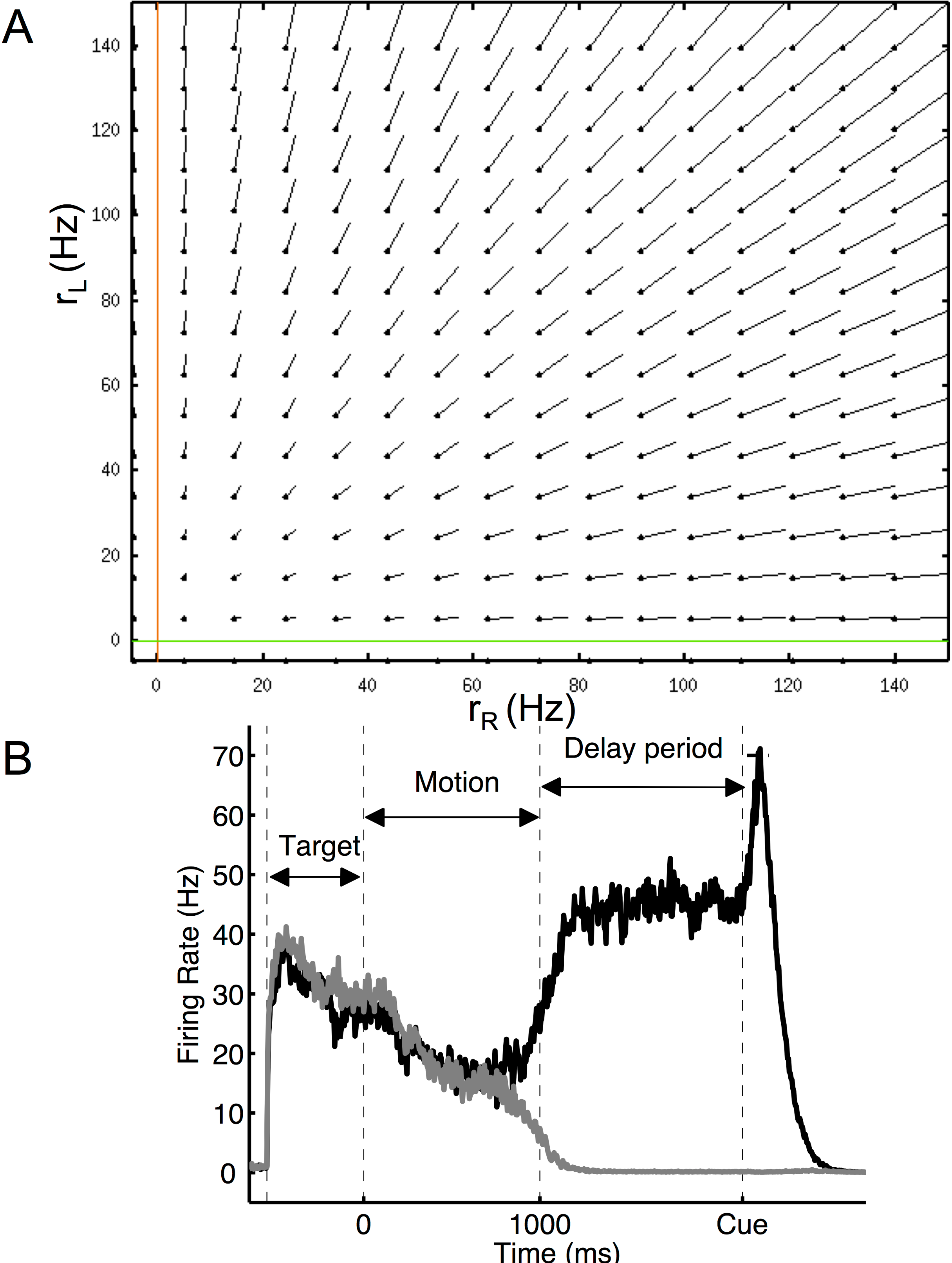

Supplement: Figure S2 — Post-decision shutdown. After the firing rate of one of the selective populations has crossed the motor threshold (70 Hz) for saccade initiation, the gains of both excitatory and inhibitory neurons are allowed to decay towards 0. (A) Phase plane at the end of a trial. The firing rate of the population selective towards leftwards () motion is plotted against the firing rate of the population selective towards rightward () motion. Orange and green curves represent nullclines: where and , respectively Only a low, symmetric attractor is present. Post-decision, trajectories start from either the upper left or the bottom right, and move towards this attractor, with local velocities shown by the arrows (B) As a result, the firing rates of both winning and losing populations are reset to baseline, before the start of the next trial, as observed in experiments. (TIF) [file pcbi.1003099.s002.tif]

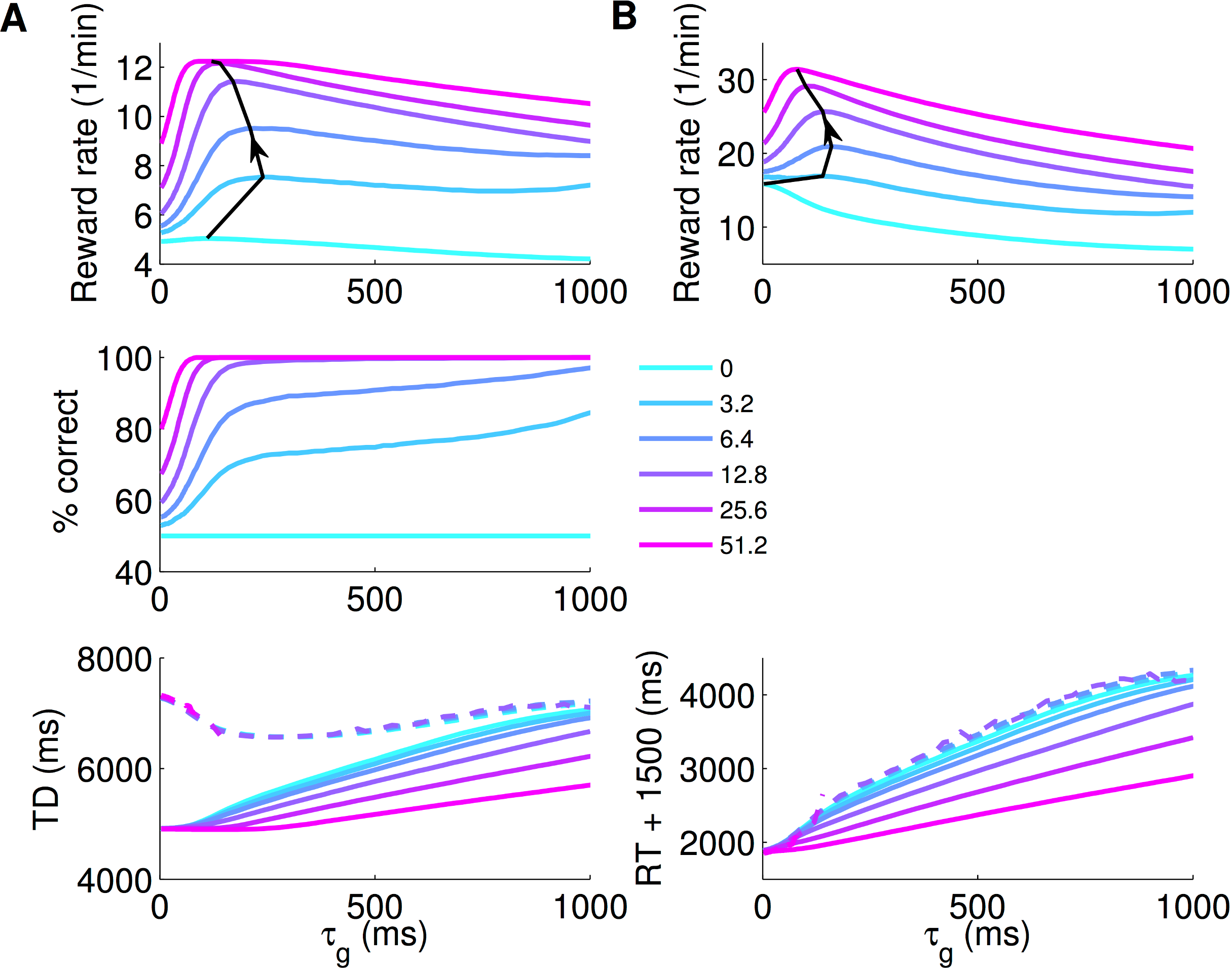

Supplement: Figure S3 — Task dependent optimal timescale of gain modulation for maximizing reward rate for individual coherences. (A) using the realistic temporal delays from Roitman and Shadlen (2002). (B) in a reaction time task with a fixed inter-trial interval (ITI). Upper, middle and lower panels show reward rate (RR), accuracy and trial duration (A) or (B). The ITI shown here is 1500 ms. Solid and dashed curves in lower panels show correct and error trials, respectively. Black arrows in upper panels show how the optimal time constant of gain modulation changes with increasing coherence. (TIF) [file pcbi.1003099.s003.tif]

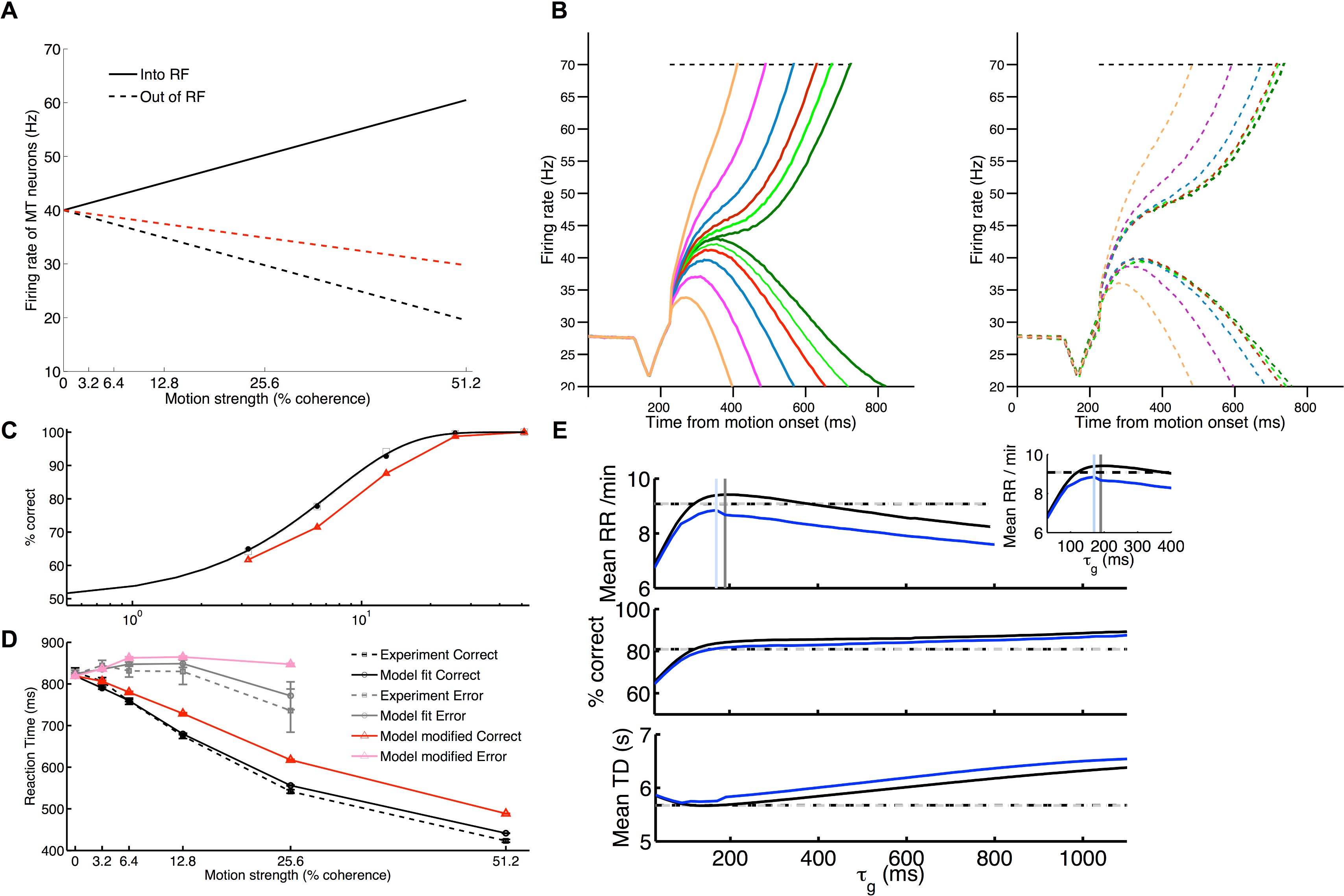

Supplement: Figure S4 — Shallower slopes in relation to motion coherence in the anti-preferred direction leads to slightly longer reaction times and poorer accuracy. (A) Timecourse of input currents for equal slopes (black) and 2 times shallower slopes (red) of input current out of the response field, in relation to motion coherence. (B) Activity timecourse of model, averaged over multiple trials, with different motion coherences for (left) equal slopes and (right) 2 times shallower slope of input current in relation to motion coherence in the anti-preferred direction. Response threshold at Hz, compare with Figure 1. (C,D) Accuracy (C) and mean RT (D) generated by model and in the experiment of [57]. The model with equal slopes (see also Figure 3) and a modified version with a 2 times shallower slope of input current in relation to motion coherence in the anti-preferred direction (red) are shown. (E) Network model performance as a function of the time constant of gain modulation using the equal slopes (black) as in the main manuscript (see Figure 10) and another with a 2 times shallower slope (blue) of input current in relation to motion coherence in the anti-preferred direction. Upper panel: mean reward rate (RR); middle panel: accuracy; lower panel: mean trial duration (TD). Dashed horizontal lines show our model's fit to the data with equal slopes and ms. Vertical lines in the upper panel show the optimal timescale of gain modulation. Inset: mean reward rate zoomed in around the optimal timescale, showing the optimal timescale is only around 20 ms shorter ms (compared to ms) when using shallower slopes for input current in relation to motion coherence in the anti-preferred direction. Shallower slopes in relation to motion coherence in the anti-preferred direction lead to a lesser discriminatory ability for the network. Thus, the firing rates for the different motion coherences ramp up/down closer together (B), resulting in poorer accuracy (C). Furthermore, the changes are more pronounce [file pcbi.1003099.s004.tif]

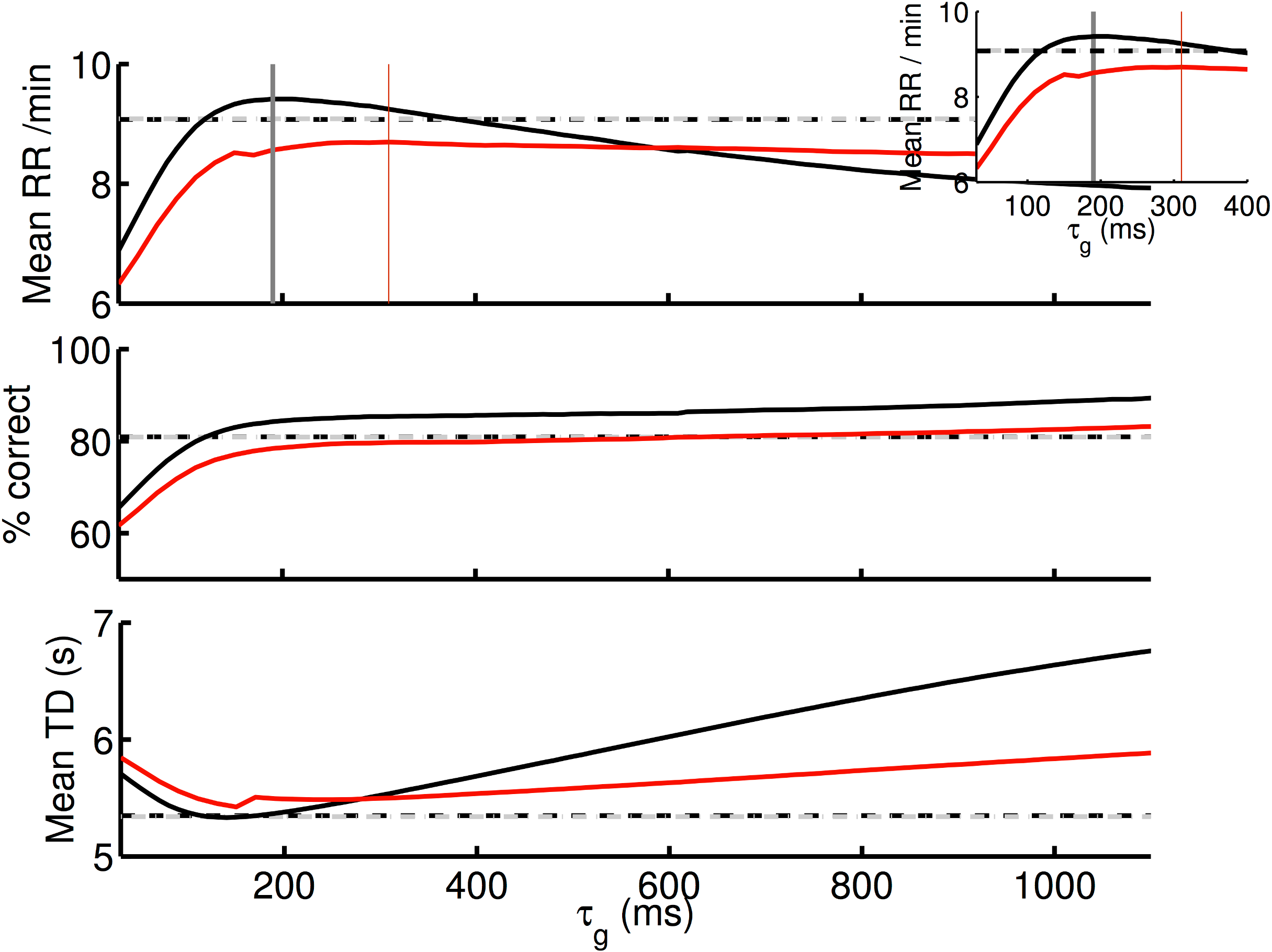

Supplement: Figure S5 — Optimal timescale of gain modulation for maximizing mean reward rate for different noise levels. Network model performance as a function of the time constant of gain modulation using the noise level (black) in the main manuscript (see Figure 10) and another with a higher (twice the standard deviation) noise level (red). Upper panel: mean reward rate (RR); middle panel: accuracy; lower panel: mean trial duration (TD). Dashed horizontal lines show our model's fit to the data with ms and noise as reported in the main manuscript. Vertical lines in the upper panel show the optimal timescale of gain modulation. Inset: mean reward rate zoomed in around the optimal timescale, showing the optimal timescale is around 200 ms longer ms (compared to ms) for a higher (twice the standard deviation) noise level. (TIF) [file pcbi.1003099.s005.tif]
