# Supplementary material for: Dynamic Excitatory and Inhibitory Gain Modulation Can Produce Flexible, Robust and Optimal Decision-making
Source: PLoS Comput Biol. 2013 Jun 27;9(6):e1003099. doi: 10.1371/journal.pcbi.1003099 (PMC3694816; doi:10.1371/journal.pcbi.1003099)
Supplement: Movie S1 — Dynamics of decision-making over an entire trial. View slideshow to play. Upper and lower panels show phase-planes and activity timecourses, respectively, throughout the various epochs of a trial for coherence . The firing rate of the population selective towards leftwards () motion is plotted against the firing rate of the population selective towards rightward () motion. Orange and green curves represent nullclines: where and , respectively. The intersection of nullclines yield the steady states. Arrows denote the local velocities of trajectories. The network starts from a low symmetric attractor during the fixation period. A burst of input current at the onset of choice targets reconfigures the network such that only a single stable steady state is present, preventing decision-making. After adaptation, the network settles to a high symmetric attractor, with additional asymmetric attractors also present. At motion onset, the inhibitory gain is increased, forming an nearby unstable steady state with a lower firing rate. The network moves towards this steady state along the diagonal line (with equal firing rates), but before it can reach it and firing rates diverge, the excitatory gain is increased, raising this unstable steady state. This reproduces the “dip” phenomenon. When the input due to the motion stimulus comes into effect, the network is reconfigured initially to a symmetric stable steady state (which prevents early divergence of firing rates), and then to a symmetric unstable steady state with a firing rate that is higher than the adapted target firing rate. This unstable steady state becomes more unstable (nullclines come closer together) as both gains increase dynamically. Consequently, the firing rates of the competing selective populations diverge and ramp up (down) more and more quickly towards the winning (losing) ‘choice’ attractor. The motor/response threshold of 70 Hz is crossed prior to the network reaching the corresponding ‘choice’ attractor, a [file pcbi.1003099.s006.pptx]

## Slide 1
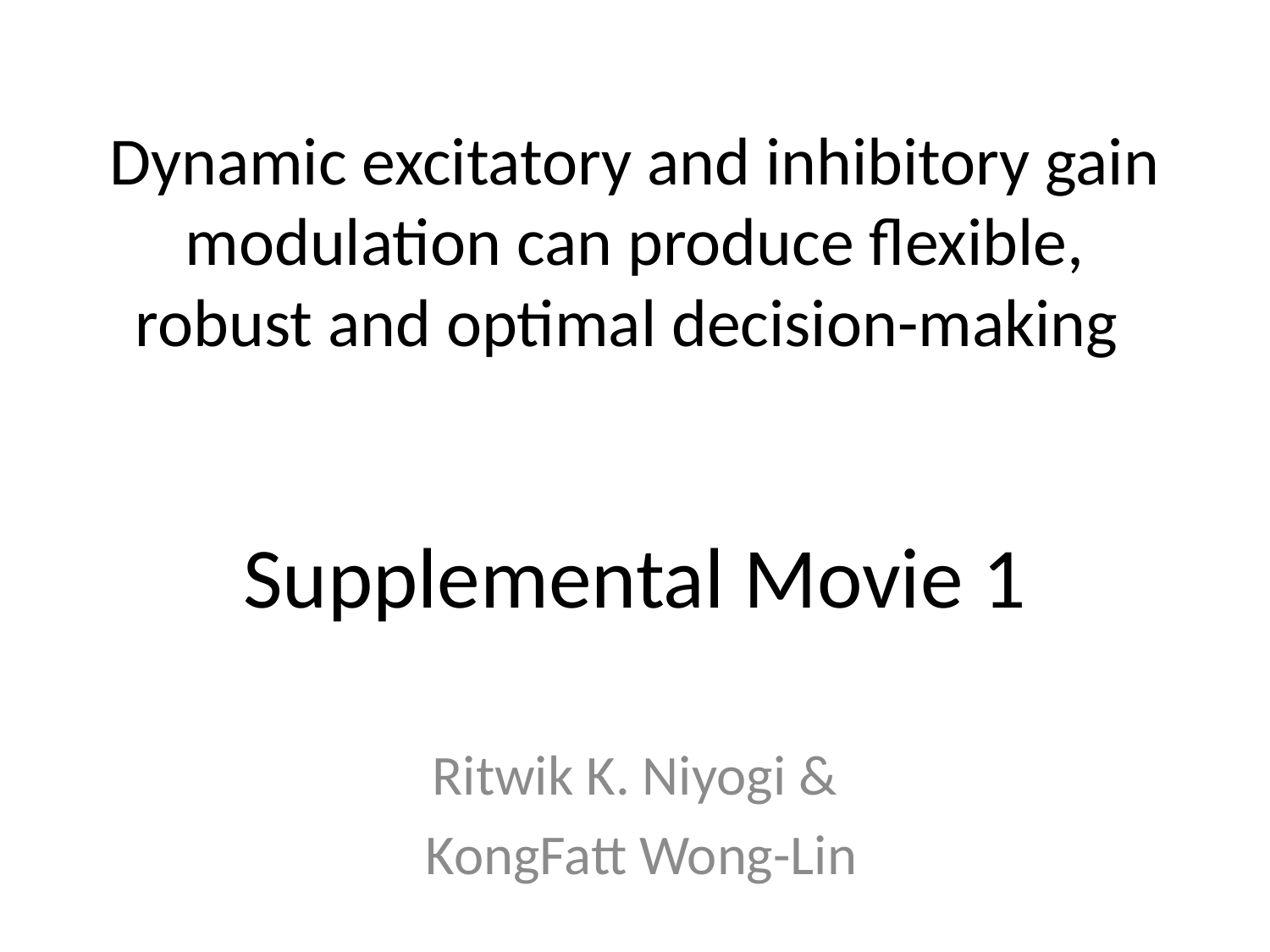

# Dynamic excitatory and inhibitory gain modulation can produce flexible, robust and optimal decision-making Supplemental Movie 1
Ritwik K. Niyogi &
 KongFatt Wong-Lin

## Slide 2
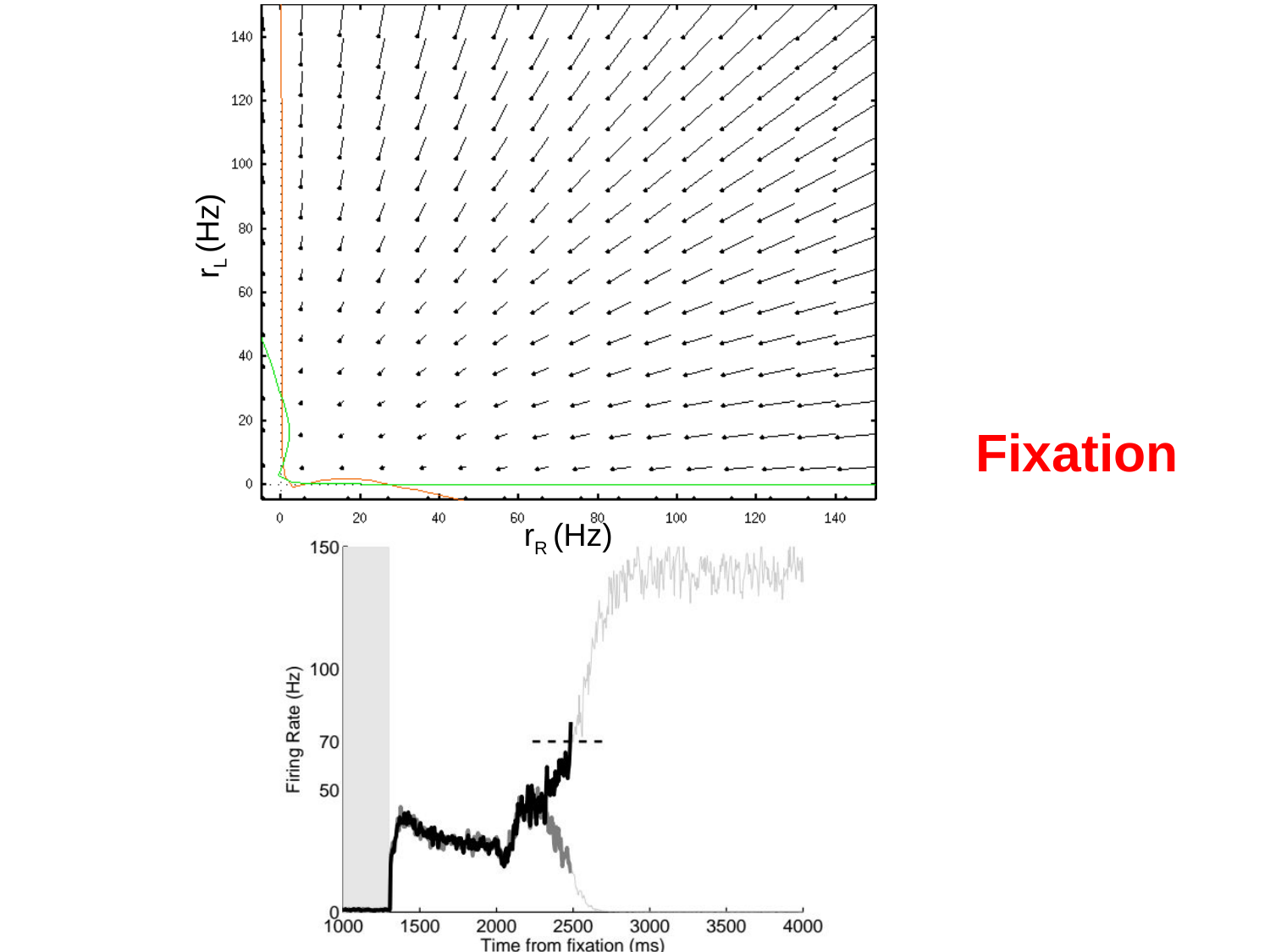

rL (Hz)
Fixation
rR (Hz)

## Slide 3
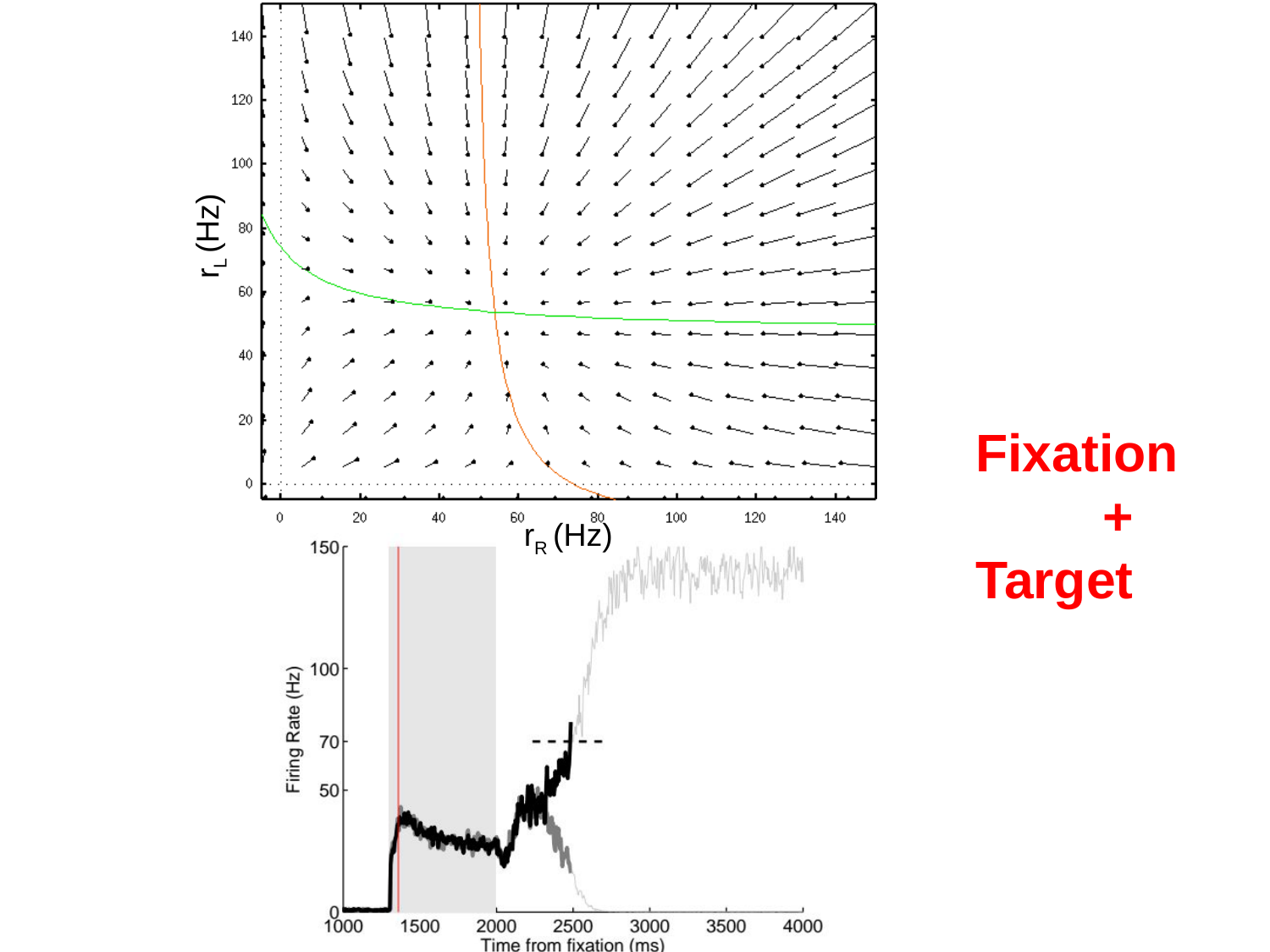

rL (Hz)
Fixation	+
Target
rR (Hz)

## Slide 4
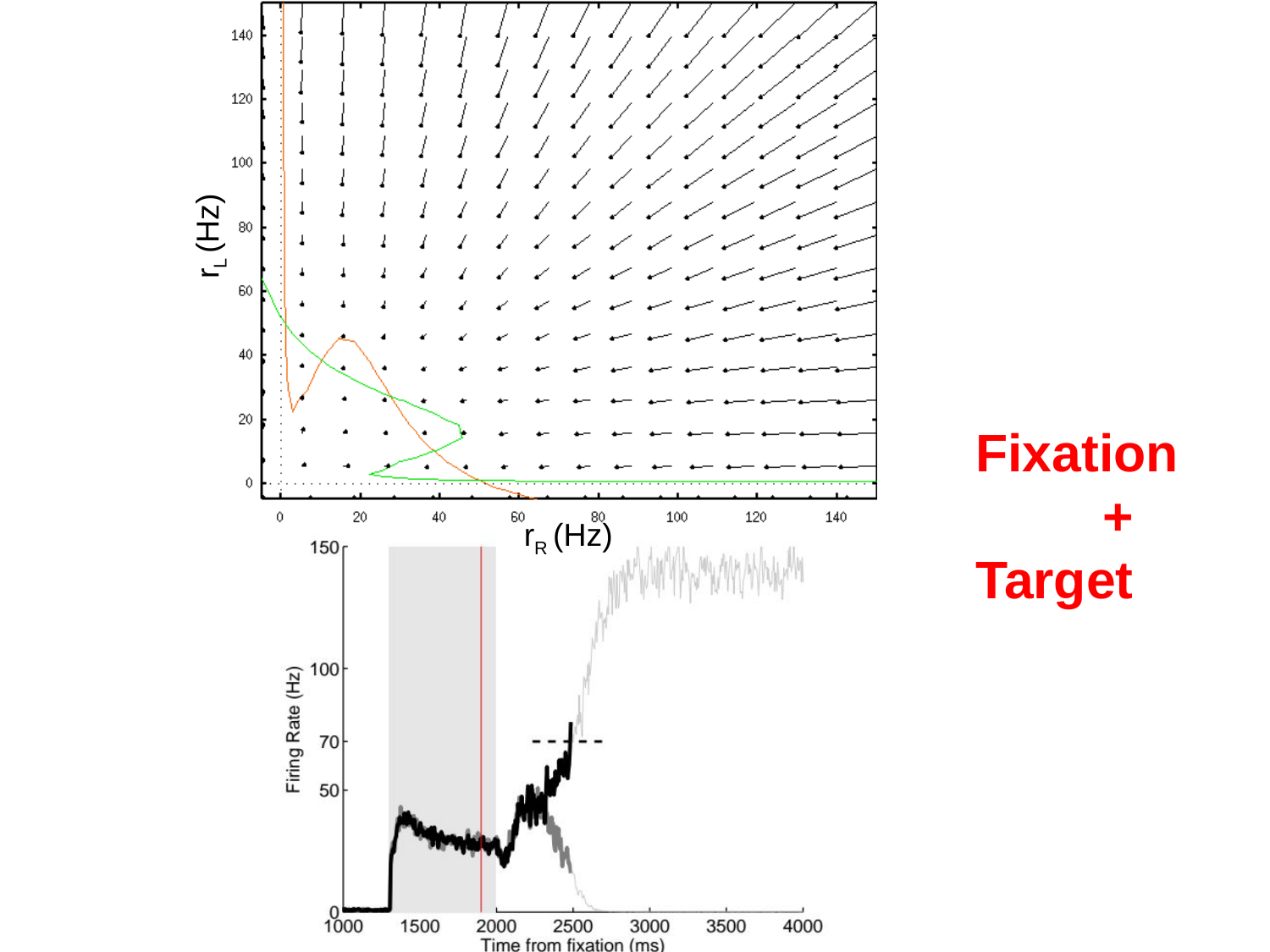

rL (Hz)
Fixation	+
Target
rR (Hz)

## Slide 5
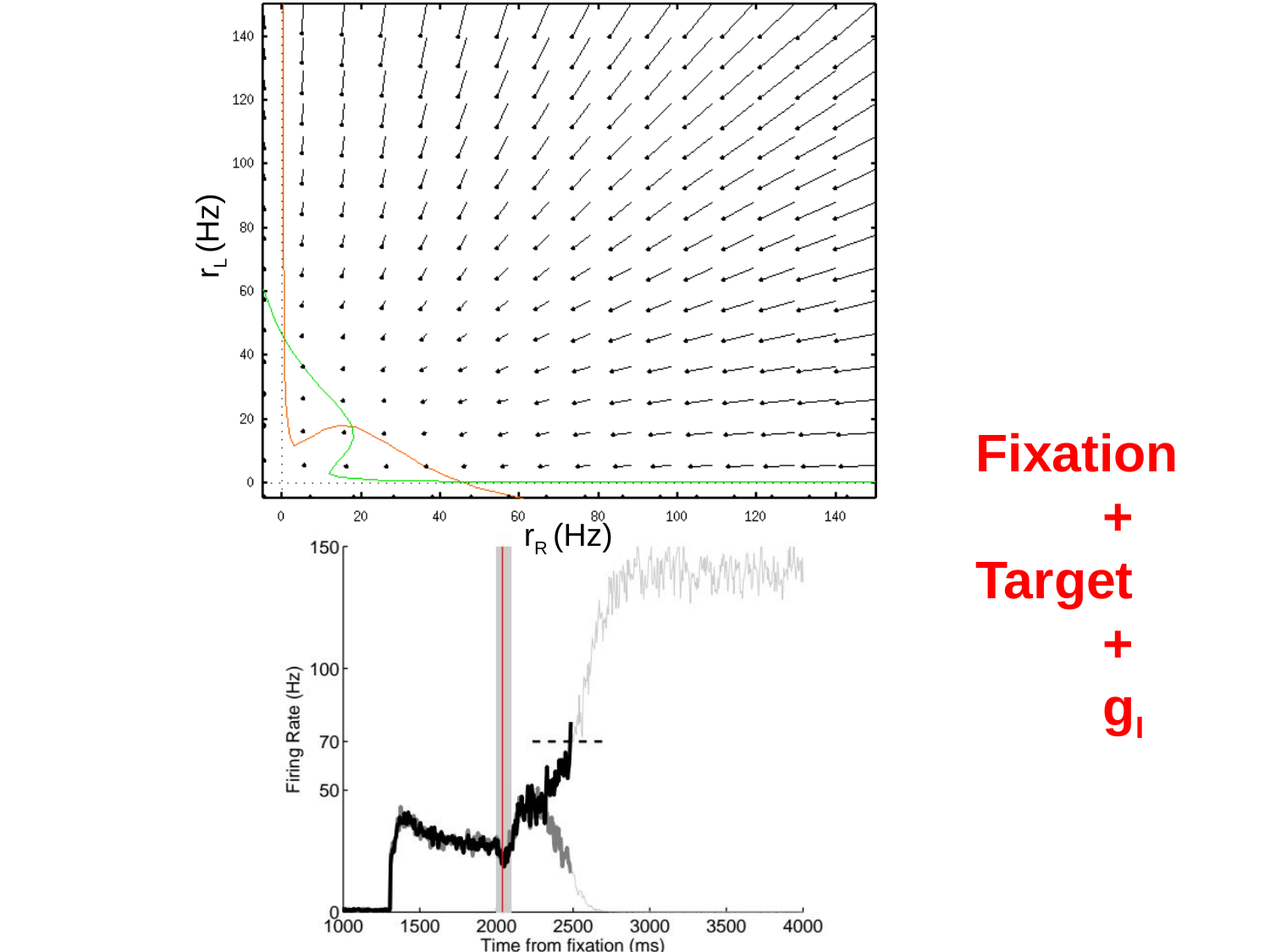

rL (Hz)
Fixation
	+
Target
	+
	gI
rR (Hz)

## Slide 6
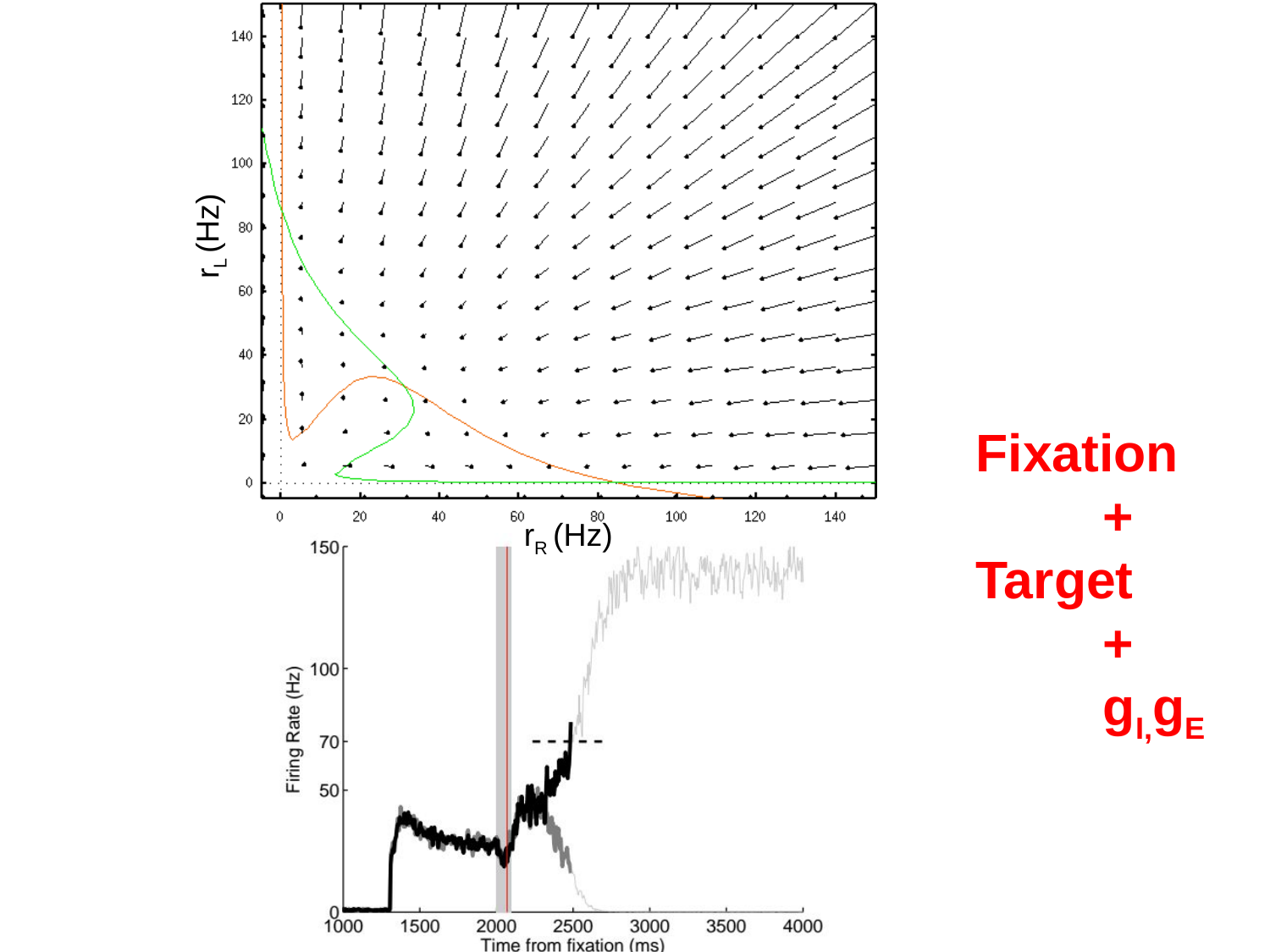

rL (Hz)
Fixation	+
Target
	+
	gI,gE
rR (Hz)

## Slide 7
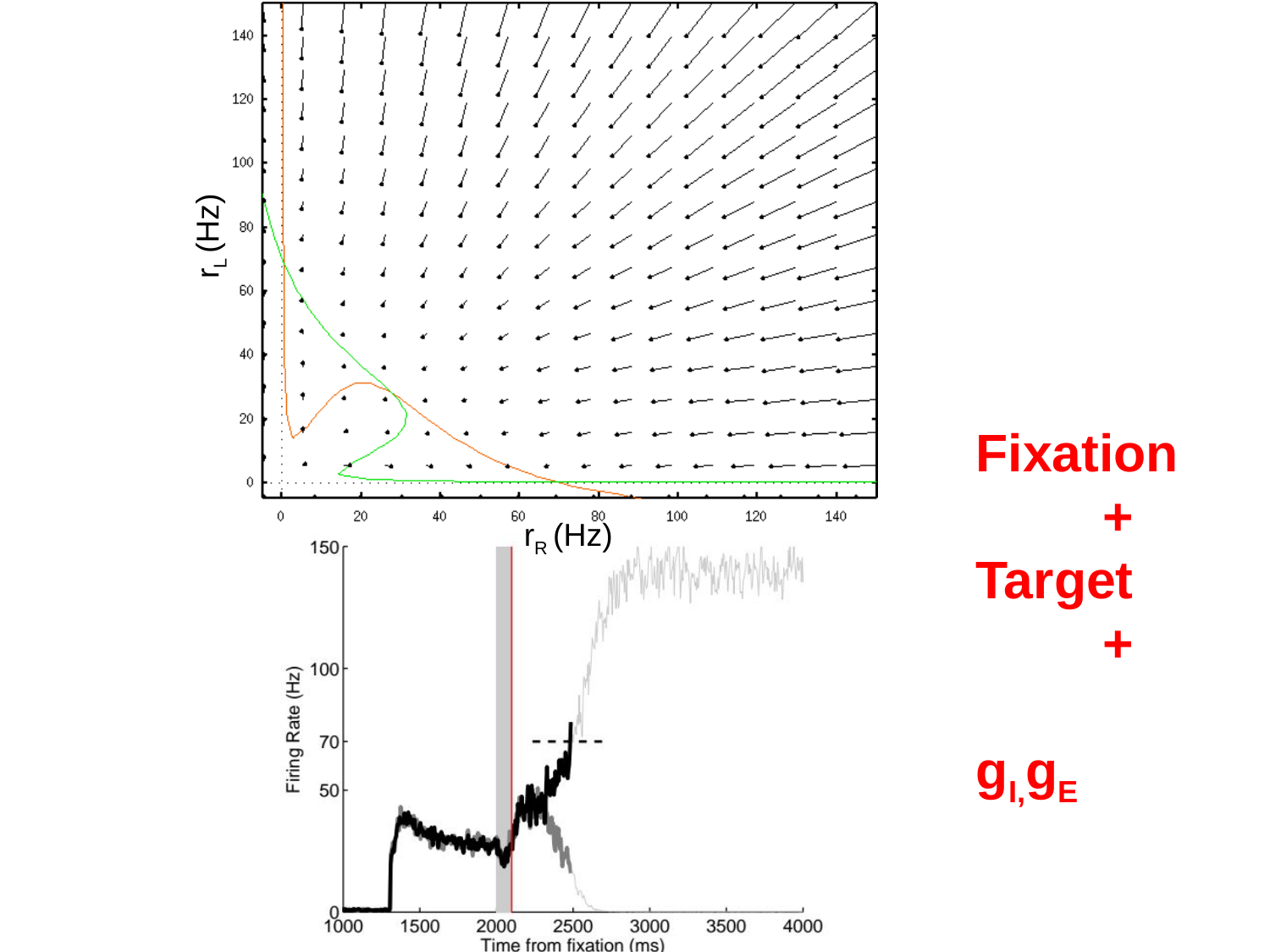

rL (Hz)
Fixation	+
Target
	+
	gI,gE
rR (Hz)

## Slide 8
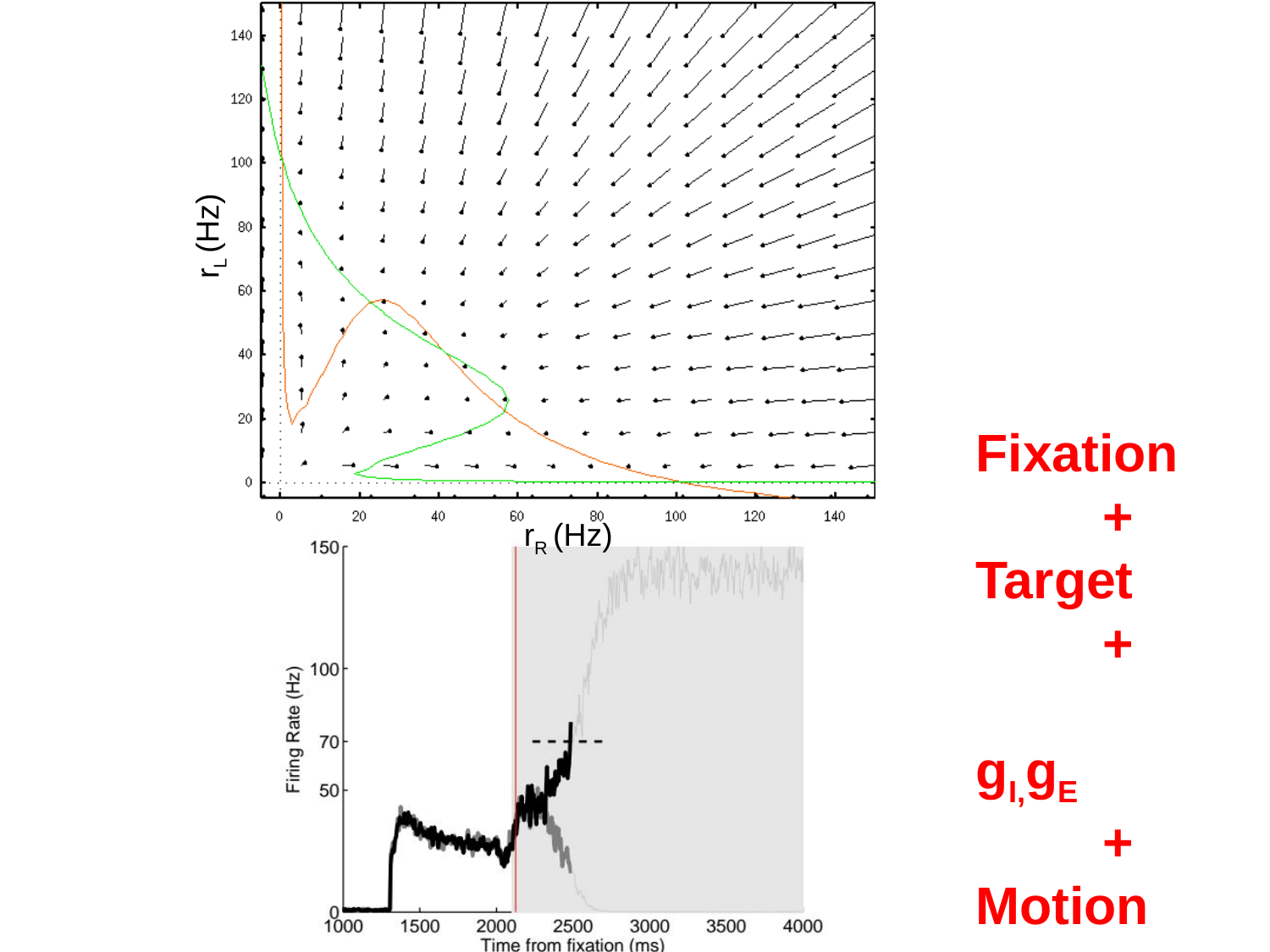

rL (Hz)
Fixation	+
Target
	+
	gI,gE
	+
Motion
rR (Hz)

## Slide 9
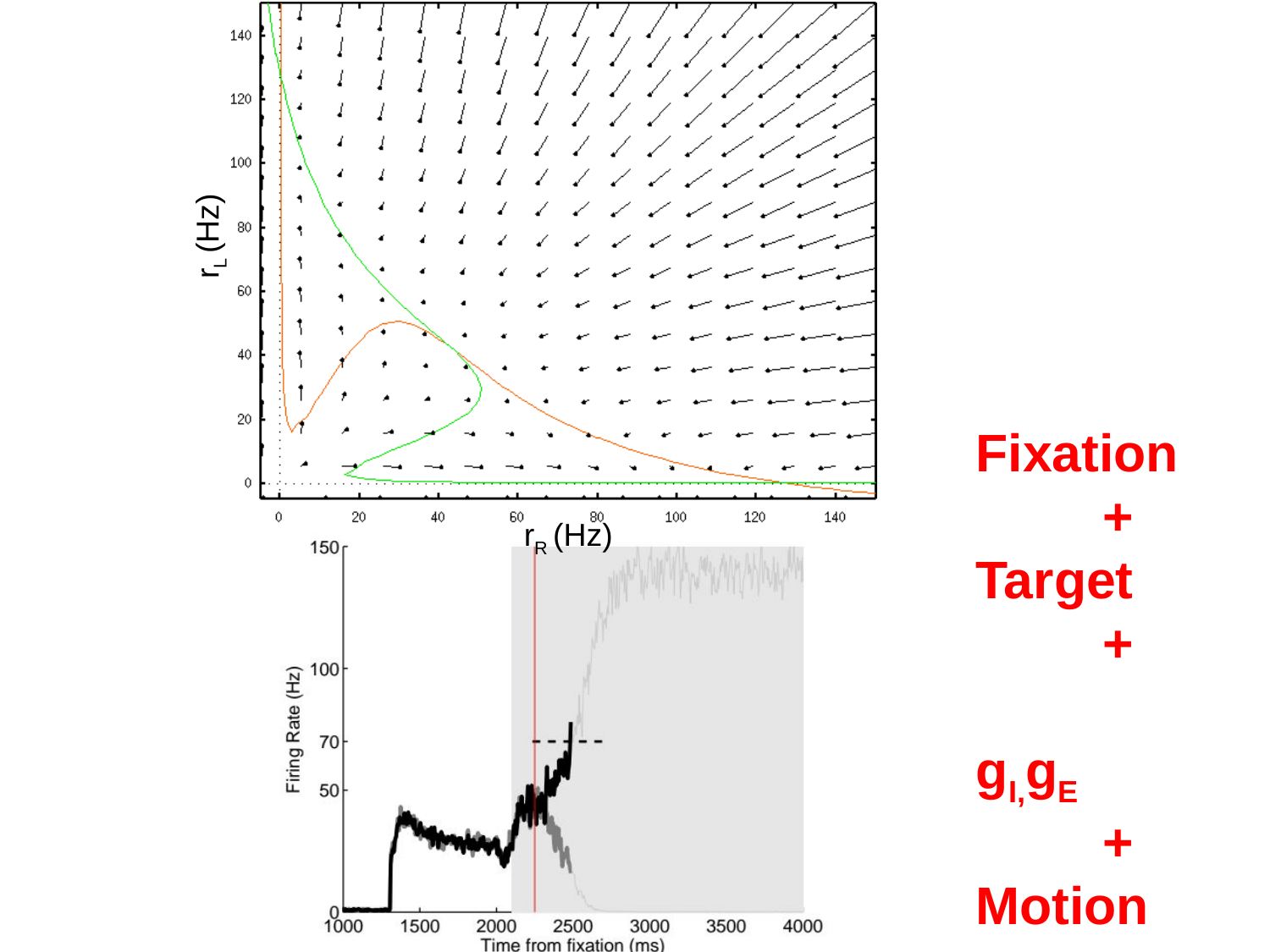

rL (Hz)
Fixation	+
Target
	+
	gI,gE
	+
Motion
rR (Hz)

## Slide 10
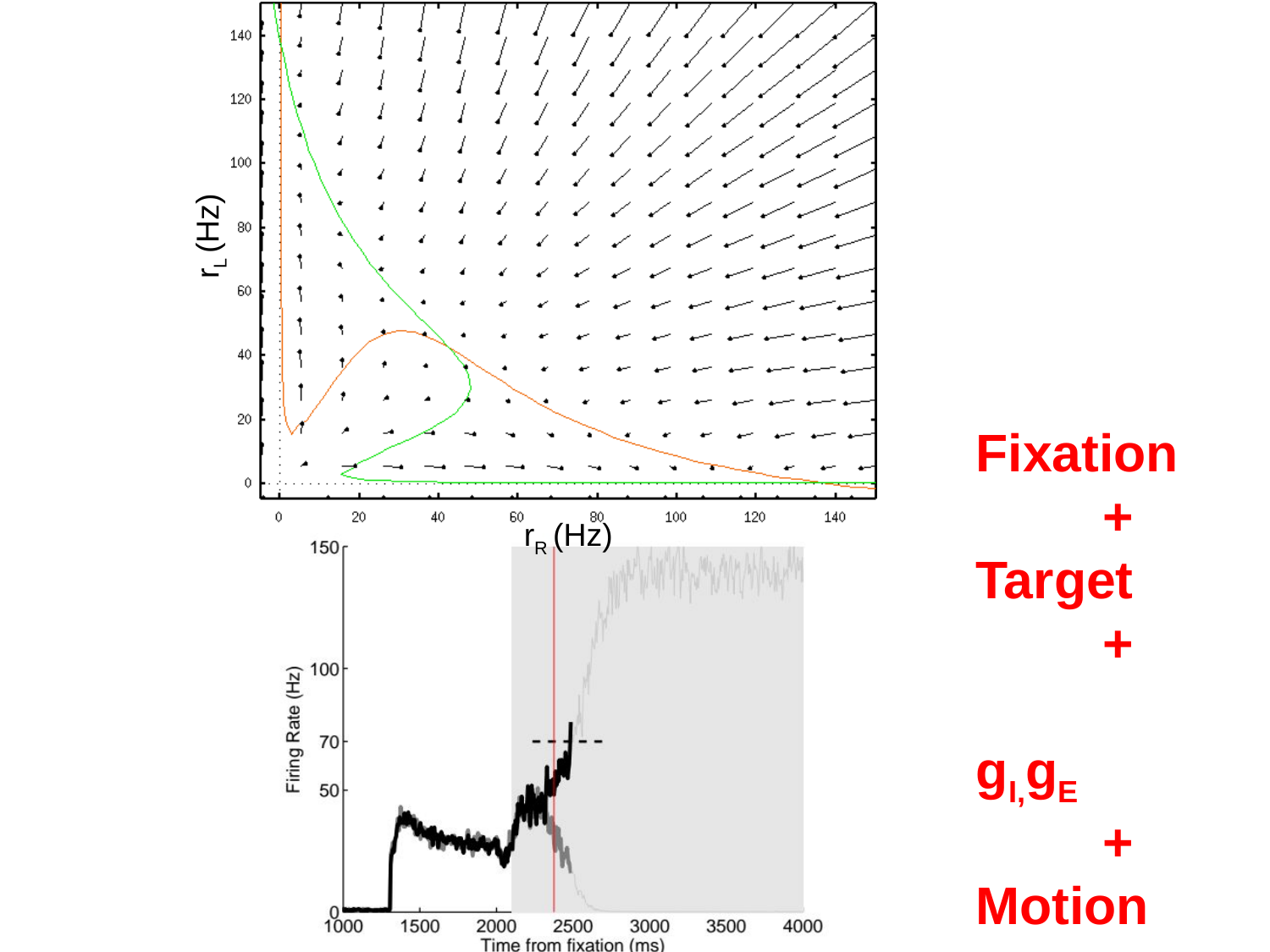

rL (Hz)
Fixation	+
Target
	+
	gI,gE
	+
Motion
rR (Hz)

## Slide 11
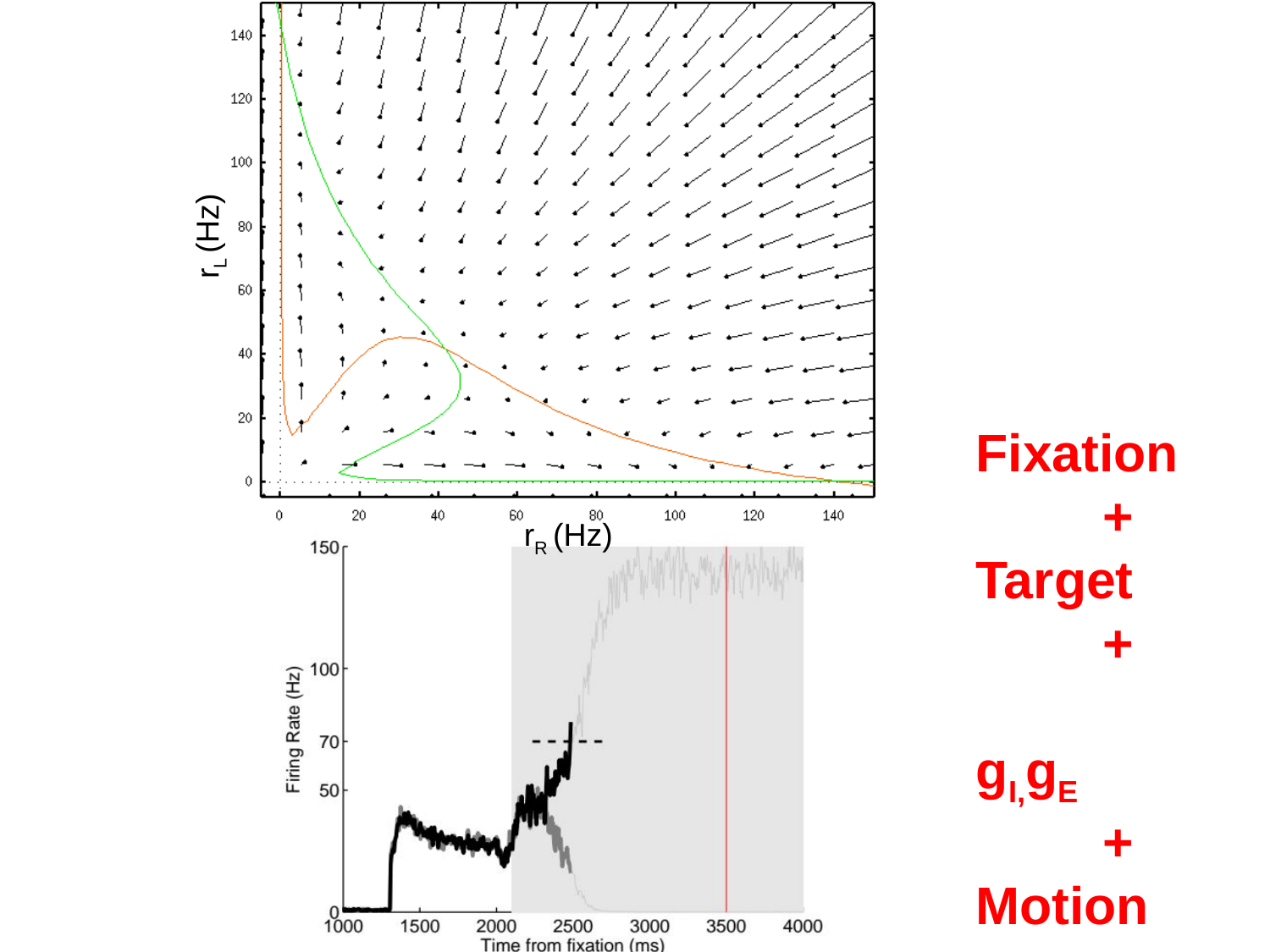

rL (Hz)
Fixation	+
Target
	+
	gI,gE
	+
Motion
rR (Hz)
